# The HIV-1 integrase-LEDGF allosteric inhibitor MUT-A: resistance profile, impairment of virus maturation and infectivity but without influence on RNA packaging or virus immunoreactivity

**DOI:** 10.1186/s12977-017-0373-2

**Published:** 2017-11-09

**Authors:** Céline Amadori, Yme Ubeles van der Velden, Damien Bonnard, Igor Orlov, Nikki van Bel, Erwann Le Rouzic, Laia Miralles, Julie Brias, Francis Chevreuil, Daniele Spehner, Sophie Chasset, Benoit Ledoussal, Luzia Mayr, François Moreau, Felipe García, José Gatell, Alessia Zamborlini, Stéphane Emiliani, Marc Ruff, Bruno P. Klaholz, Christiane Moog, Ben Berkhout, Montserrat Plana, Richard Benarous

**Affiliations:** 1 Biodim Mutabilis, 93230 Romainville, France; 2 INSERM, U1016, Institut Cochin, Paris, France; 3 CNRS, UMR8104, Paris, France; 4 University Paris Descartes, Sorbonne Paris Cité, Paris, France; 5 Laboratory of Experimental Virology, Department of Medical Microbiology, Center for Infection and Immunity Amsterdam (CINIMA), Academic Medical Center, University of Amsterdam, Amsterdam, The Netherlands; 6 Centre for Integrative Biology, IGBMC, CNRS, INSERM, University of Strasbourg, Strasbourg, France; 7 AIDS Research Group, IDIBAPS, Hospital Clinic, Barcelona, Spain; 8 INSERM U1109, Strasbourg, France; 9 CNRS, UMR7212, INSERM U944, Université Paris Diderot, Conservatoire National des Arts et Métiers, Paris, France; 10 Present Address: 19 rue de Croulebarbe, 75013 Paris, France

**Keywords:** HIV-1, Integrase, LEDGF, Allosteric integrase inhibitor, LEDGIN, INLAI, Immunoreactivity

## Abstract

**Background:**

HIV-1 Integrase (IN) interacts with the cellular co-factor LEDGF/p75 and tethers the HIV preintegration complex to the host genome enabling integration. Recently a new class of IN inhibitors was described, the IN-LEDGF allosteric inhibitors (INLAIs). Designed to interfere with the IN-LEDGF interaction during integration, the major impact of these inhibitors was surprisingly found on virus maturation, causing a reverse transcription defect in target cells.

**Results:**

Here we describe the MUT-A compound as a genuine INLAI with an original chemical structure based on a new type of scaffold, a thiophene ring. MUT-A has all characteristics of INLAI compounds such as inhibition of IN-LEDGF/p75 interaction, IN multimerization, dual antiretroviral (ARV) activities, normal packaging of genomic viral RNA and complete Gag protein maturation. MUT-A has more potent ARV activity compared to other INLAIs previously reported, but similar profile of resistance mutations and absence of ARV activity on SIV. HIV-1 virions produced in the presence of MUT-A were non-infectious with the formation of eccentric condensates outside of the core. In studying the immunoreactivity of these non-infectious virions, we found that inactivated HIV-1 particles were captured by anti-HIV-specific neutralizing and non-neutralizing antibodies (b12, 2G12, PGT121, 4D4, 10-1074, 10E8, VRC01) with efficiencies comparable to non-treated virus. Autologous CD4^+^ T lymphocyte proliferation and cytokine induction by monocyte-derived dendritic cells (MDDC) pulsed either with MUT-A-inactivated HIV or non-treated HIV were also comparable.

**Conclusions:**

Although strongly defective in infectivity, HIV-1 virions produced in the presence of the MUT-A INLAI have a normal protein and genomic RNA content as well as B and T cell immunoreactivities comparable to non-treated HIV-1. These inactivated viruses might form an attractive new approach in vaccine research in an attempt to study if this new type of immunogen could elicit an immune response against HIV-1 in animal models.

**Electronic supplementary material:**

The online version of this article (10.1186/s12977-017-0373-2) contains supplementary material, which is available to authorized users.

## Background

The integration of a DNA copy of the HIV RNA genome into host chromatin is a crucial step of HIV replication [1]. The HIV-1 pre-integration complex is tethered to the host chromosome via the cellular co-factor lens epithelium-derived growth factor (LEDGF/p75) [2], together with the involvement of the capsid binding protein CPSF6 [3]. LEDGF/p75 is a chromatin-bound protein that interacts with HIV-1 Integrase (IN) via its C-terminal IN binding domain (IBD) [4, 5]. A new class of IN-inhibitors was designed that prevents this IN-LEDGF/p75 interaction, named first LEDGINs [6], then ALLINIs [7] for Allosteric IN inhibitors, NCINIs [8–10] for non catalytic IN inhibitors, MINIs for Multimerization Integrase Inhibitors [11] or INLAIs for Integrase-LEDGF allosteric inhibitors [12]. Since their first description by the group of Zeger Debyser [6], there is not yet a consensus name or acronym for this new class of IN inhibitors; we chose in this report the acronym INLAI as a generic name for these inhibitors, which has the advantage to recall the dual mechanism of action of these inhibitors: inhibition of the IN-LEDGF/p75 interaction and induction of an allosteric conformational change and multimerization of IN.

INLAIs are allosteric IN inhibitors that bind to the LEDGF binding pocket of IN and are fully active on HIV-1 resistant to INSTIs [6–10, 12–14]. From a chemical point of view, all INLAIs described up to date share a common motif composed of a tert-butylether and a carboxylic acid group that can be linked to different scaffolds, quinoline, naphthyl, phenyl or pyrimidine [6–10, 12–14]. INLAIs have a dual antiretroviral (ARV) activity at two different steps of the HIV-1 replication cycle: Inhibition of the LEDGF/p75-IN interaction accounts for an “early” block of HIV-1 replication at integration, but the major impact of INLAIs is during virus maturation or the “late” phase, leading to the production of normal CA-p24 amounts of non-infectious virus. This late effect on virus maturation is linked to INLAI-promoted IN multimerization [9, 12–15].

HIV-1 virions produced in the presence of INLAIs are non-infectious and contain eccentric condensates outside of the cores as shown by electron microscopy [9, 14, 15]. However, using HIV-1 produced in the presence of the quinoline INLAI compound BI-D (developed by Boehringer Ingelheim), we recently described that a wild-type level of HIV-1 genomic RNA is packaged in these virions in a dimeric state, and the tRNA^lys3^ primer for reverse transcription was properly placed on the genomic RNA and could be extended ex vivo. In addition, RT enzyme extracted from these virions was fully active although these virions were unable to complete reverse transcription in target cells [9]. Fontana et al. [16] found that INLAIs block ribonucleoprotein complex packaging inside viral cores leading to the formation of “eccentric condensates” with high Nucleocapsid (NC) content outside the core. Kessl et al. [17] showed recently that IN directly binds the viral RNA genome in virions and that ALLINIs impair IN binding to viral RNA in wild-type virions. These INLAI-inactivated virions were able to infect target cells, but the subsequent reverse transcription step in target cells was blocked [9, 15]. Vranckx et al. [18] have shown that LEDGF/p75 depletion hampers HIV-1 reactivation in cell culture, and they demonstrated that LEDGINs relocate and retarget HIV integration, resulting in a HIV reservoir that is refractory to reactivation by different latency-reversing agents.

Several attempts have been made to produce inactivated SIV vaccines by treatment with denaturing and inactivating agents such as zinc chelators (2,2′-dithiobisbenzamide (DIBA)), heat, UV or cross-linking agents such as psoralen in non-human primate studies. But none of these attempts has shown significant efficacy in preventing SIV infection or in controlling virus replication and delaying progression of disease (for review [19]). HIV-1 inactivated by similar denaturing methods has also been used in dendritic cell-based therapeutic vaccination, but did not yet yield convincing results [20, 21]. INLAI-inactivated HIV-1 particles have remarkably conserved many properties of “native” non-treated HIV-1 particles, but are based on a new ARV drug class under preclinical development and not on a denaturing chemical. In front of the persisting bottlenecks in identifying viable HIV vaccine candidates, we explored the main B and T cell immunoreactivities of INLAI-inactivated virus particles to study whether they could be considered as a new type of immunogen able to elicit immune response against HIV-1 in animal models.

**Fig. 1:**
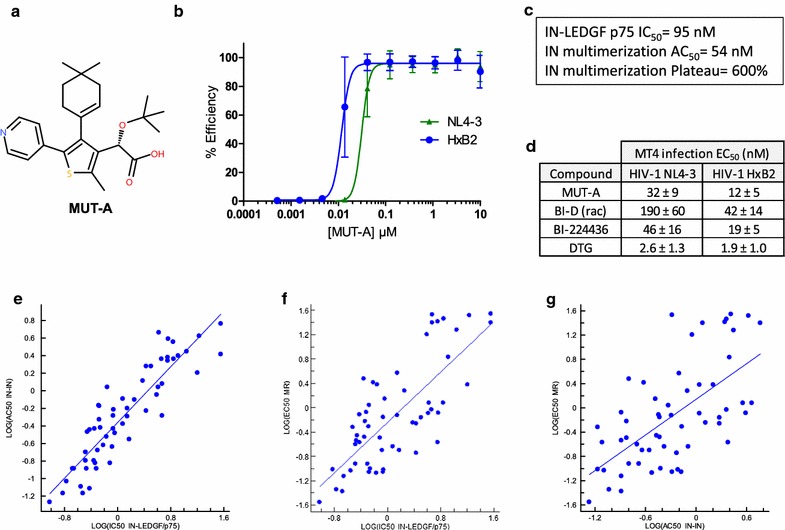
Structure, biochemical and antiretroviral activities of MUT-A. **a** Chemical structure of MUT-A. **b** Titration of MUT-A ARV activity on HIV-1 NL4-3 and HxB2 strains in MT4 cell infection assay. **c** Biochemical activities of MUT-A on IN-LEDGF/p75 interaction (IC_50_) and of IN multimerization (activation concentration AC_50_ and maximum signal increase plateau). **d** EC_50_ of ARV activities of MUT-A and references HIV-1 NL4-3 and HxB2 strains in MT4 cell infection assay. DTG: INSTI Dolutegravir. **e**–**g** Correlations between IN multimerization (IN–IN) AC_50_ and IN-LEDGF/p75 IC_50_ (**e**), ARV EC_50_ and IN-LEDGF IC_50_ (**f**), ARV EC_50_s and IN–IN AC_50_s (**g**) of MUT-A compound series

Here we describe the new INLAI compound MUT-A. MUT-A shares with all previously described INLAIs a tert-butylether motif linked to a carboxylic acid group, but it is composed of an original scaffold, a 5-atom thiophene ring (Fig. 1). We studied the ARV activity of MUT-A, its resistance profile, its influence on viral RNA packaging and the B-cell and T-cell immunoreactivities of MUT-A-inactivated HIV-1.

## Methods

### Cell culture

SupT1 or MT4 T cells were cultured in advanced RPMI 1640 medium (Gibco) supplemented with 10% (v/v) heat-inactivated fetal bovine serum (FBS, Gibco), 2 mM l-glutamine (Gibco), 15 µg/mL streptomycin and 15 units/mL penicillin at 37 °C and 5% CO_2_. Human embryonic kidney (HEK) 293T cells were grown in DMEM (Gibco) supplemented with 10% (v/v) heat-inactivated FBS (Gibco) and 1× minimum essential medium non-essential amino acids (MEM NEAA, Gibco) at 37 °C and 5% CO_2_.

### Virus strains and recombinant molecular clones

HIV-1 NL4-3, HxB2 molecular clones were obtained from the NIH AIDS Research and Reference Reagent Program and SIVmac239 was a gift of R. Desrosiers. The SpeI-SalI fragment from pNL4-3 containing the full *pol* gene was cloned into the pUC18 plasmid. In vitro mutagenesis was performed with the PfuTurbo (Agilent) and specific sets of primers to engineer the single mutants in *integrase* (Y99H, A128T, L102F, H171Q/T, T174I, N222K). The mutated fragment was validated by sequencing (Eurofins MWG Operon) and cloned back into pNL4-3 to generate a HIV-1 mutant molecular clone.

### Compound

Optimization of our small molecule series led to compound MUT-A, one of the most active molecules. MUT-A was prepared as described in details in Additional file 1 and in a patent application [22], according to the example 11. The chemical identification of MUT-A and its purity were assessed by NMR spectrum (shown in Additional file 1: Fig. S1). MUT-A was dissolved in DMSO to generate a 10 mM stock solution and was added to the culture medium at a final concentration as indicated. The equivalent volume of DMSO was added to control cultures.

### HTRF^®^-based IN-LEDGF interaction assay

The IN-LEDGF HTRF^®^ assay was performed using Flag-tagged IN NL4-3 and His-tagged LEDGF/p75 as described in [12], and detailed in Additional file 1.

### HTRF^®^-based IN multimerization assay

The IN–IN HTRF^®^ assay was performed using Flag-tagged and His-tagged IN from NL4-3, as described in [12] and detailed in Additional file 1.

### Virus production and replication

The experiments with HIV-1 isolates LAI or NL4-3 were performed as described in [23] and the data presented here were collected simultaneously with those reported previously in [23] using the INLAI BI-D together with the same untreated controls. 293T cells were seeded in T75 culture flasks, cultured to 50–70% confluency and transfected with 20 μg pLAI or pNL4-3 DNA plasmid that encodes the wt HIV-1 LAI or HIV-1 NL4-3 isolates respectively using Lipofectamine2000 (Invitrogen). MUT-A was added 6 h after transfection. The culture supernatant was harvested 48 h after transfection and used as virus stock or for viral RNA isolation. The CA-p24 level was measured by enzyme-linked immunosorbent assay (ELISA) as described previously [23]. SupT1 T cells (5 × 10^6^ cells in 5 mL) were infected with the HIV-1 LAI virus stocks (equivalent of 1 ng CA-p24). Similarly, MT4 cells (5 × 10^6^ cells in 5 mL) were infected with the HIV-1 NL4-3 virus stocks (equivalent of 1 ng CA-p24). When indicated, the culture was split and MUT-A or DMSO was added. Viral spread was monitored by measuring the CA-p24 level in the virus culture medium every 2 days. HIV-1 NL4-3 virus produced and inactivated in the presence of AT-2 was prepared as described in [24].

### HIV-1 and SIV antiviral assays in MT4 cells

MT4 cells growing exponentially at the density of 10^6^/mL were infected for 2 h with HIV-1 strain NL4-3, HxB2 or SIVmac239 (viral stock produced according to [25]) at a multiplicity of infection (MOI) of 0.006 and 0.01, respectively. The cells were washed with PBS and aliquoted, using 100 μL fresh complete RPMI, into 96-well white plates (Corning) in the presence of different concentrations of compounds. The effective concentration of compound required to inhibit 50% (EC_50_) of viral replication was determined, after 5 or 7 days for HIV-1 and SIV respectively, using the CellTiter-Glo^®^ luminescent reagent (Promega) to quantify cell viability.

### Viral resistance selection

Resistance selection was carried out by serial passages of infection at suboptimal compound concentrations. MT4 cells were seeded in 96-well culture plates at a density of 1 × 10^5^ cells/well in 200 µL of culture medium. HIV-1 NL4-3 was used at 0.005 MOI. Compounds were added at final concentrations corresponding to ½ EC_50_, EC_50_ and 2× EC_50_ and the plates were done in triplicate. Every 3–4 days, measurement of the antiviral activity allowed selecting between the three successive concentrations which one to choose to go on with the next passage. The cytopathic effect induced by HIV-1 was used to follow the progression of the infection. Briefly, 50 µL of the cell culture from the passage plate were mixed with 50 µL CellTiter-Glo^®^ reagent to quantify cell viability. The concentration corresponding to the lower protection (0–25%) at the highest concentration was chosen to proceed. All the content from the chosen well was transferred to a 1.5 mL microtube and centrifuged for 3 min at 3000 rpm at room temperature. 30 µL of the supernatant were then used to infect three wells seeded with fresh cells and twofold serial dilutions of the compound. Successive viral passages were obtained by repeating this procedure.

### Clonal sequencing of viral DNA

For clonal sequencing of viral passages, total DNA was extracted from infected cells using the QIAamp DNA blood minikit (Qiagen). A 980-bp viral DNA fragment containing the entire IN orf, spanning from nucleotide 4176 in the *pol* gene to nucleotide 5154 in the *vif* gene, was amplified by PCR (nucleotide numbering according to pNL4-3 molecular clone sequence (Accession number AF324493)). A 610-bp viral DNA fragment spanning from nucleotide 2588–3199 in the *pol* gene was also amplified by PCR. This fragment contains the region of RT sensitive to mutation under the pressure of selection. DNA extracted from non-infected cells was used as a negative control in PCR experiments to ensure there was no cross contamination. PCR products were purified using EZ-10 Spin Column PCR Purification kit (BioBasic) and cloned into the pCR2.1^®^ using the TA cloning kit (Invitrogen). The IN or RT gene from 30 to 40 single clones was sequenced by Eurofins sequencing service (Eurofins MWG Operon) using specific primers. The sequence was compared to the wild-type NL4-3 region using NCBI BLAST alignment engine and amino acid substitutions were identified.

### B-cell immunoreactivity

B-cell immunoreactivity of HIV-1 NL4-3 virus produced in the absence or presence of MUT-A was studied by virus immunocapture assays as described earlier [26], using the panel of anti-HIV-1 neutralizing and non-neutralizing antibodies [27–35] listed in Fig. 6b. This assay measures the concentration of whole native virus particles captured by antibodies (Abs) coated onto 96-well plates (Maxisorp, Nunc) as previously described [26, 36, 37]. Briefly, HIV particles were incubated in Ab-coated ELISA plates for 1 h. Unbound virus was removed by washing with PBS containing 10% fetal calf serum. Virus captured by Abs was lysed with 10% NP-40 and quantified by dosage of CA-p24 by ELISA (Innogenetics/Ingen).

### Patients and samples

Samples of EDTA-anticoagulated venous blood samples were obtained from chronic asymptomatic HIV-1-infected patients with baseline CD4^+^ T-cell counts > 450 cells/mm^3^, and plasma viral load (pVL) < 50 HIV-1 RNA copies/mL, who were on antiretroviral therapy (ART). All the subjects participating in the study were recruited at the Service of Infectious Diseases and AIDS Unit of the Hospital Clinic from Barcelona (Spain). All the individuals gave informed written consent and this study was reviewed and approved by the Institutional Ethical Committee board of Hospital Clinic (Barcelona, Spain).

### Generation of monocyte-derived dendritic cells

Generation of monocyte-derived dendritic cells (MDDC) was performed as previously described [38]. Essentially, peripheral blood mononuclear cells (PBMC) were isolated immediately after venous extraction by using a standard Ficoll gradient. Cells were processed immediately after isolation. To obtain human monocytes, PBMC were incubated in plastic plates (2 h at 37 °C) in a humidified atmosphere with 5% CO_2_ in MDDC medium (serum-free XVIVO-15 medium, Lonza) supplemented with 1% autologous serum, gentamicin (Braun Medical) and fungizone (amphotericin B, Bristol-Myers Squibb) and 1 µM zidovudine (Retrovir from GlaxoSmithKline) to avoid HIV replication. To obtain immature MDDC, adherent cells were washed four times with pre-warmed MDDC medium and then cultured for 5 days in the presence of 1000 U/mL each of recombinant human IL-4 (Strathmann Biotec AG) and recombinant human GM-CSF (Peprotech) on day 0 and 2. Immature MDDC in fresh MDDC medium, IL-4 and GM-CSF (1000 U/mL each) were exposed to NL4-3 virus either treated with DMSO, inactivated with MUT-A at 1 µM (1 or 5 µg/mL of HIV Gag CA-p24), inactivated with AT-2 1 µg/mL, and controls (SEA (Staphylococcus Enterotoxin A) 100 pg/mL). To obtain mature MDDC, a cocktail of recombinant human cytokines containing TNF-α, IL-6 (1000 IU/mL each, Strathmann Biotec AG), IL-1β (300 IU/mL, Strathmann Biotec AG) and PGE2 (1 mg/mL, Pfizer) was added at 2 h post-exposure, and the mixture was incubated for 48 h.

### Autologous co-cultures

As a source of enriched T cells we employed autologous fresh PBMC depleted of monocytes after adherence to plastic as indicated above for the generation of MDDC. These monocyte-depleted lymphocytes were washed and resuspended in serum-free XVIVO-10 medium and labelled with CFSE following the instructions of the manufacturer (CellTrace CFSE cell proliferation kit, Molecular Probes). Autologous virus-exposed and matured MDDC were washed and resuspended in XVIVO-10 and co-cultured with autologous fresh CFSE-labelled lymphocytes in a final volume of 0.2 mL in XVIVO-10 medium supplemented with 1 μM zidovudine to impede possible replication of endogenous HIV-1. The contribution of MDDC alone and monocyte-depleted PBMC (lymphocytes) alone was determined as negative controls of proliferation and cytokine secretion. The co-cultures were done in triplicates at 37 °C in a humidified atmosphere of air with 5% CO_2_.

### Assessment of T cell proliferation and flow cytometry

After 6–7 days, proliferating CD3^+^CD4^+^ and CD3^+^CD8^+^ T cells were determined by direct staining with mAbs conjugated with a-CD3-Per-CP and a-CD8-PE. Mouse Ig isotypes mAbs (from BD Biosciences) conjugated with PerCP or PE were used as negative control mAbs. The stained cells were analyzed on a FACSCalibur flow cytometer (BD Biosciences). T cell populations were selected by forward and side light-scatter parameters and sub-gated for CD4 or CD8 expression. Cells that proliferated after the co-culture had lower intensity of CFSE (CFSE^low^) in comparison with basal conditions. T cell specific proliferation was expressed as the percentage of CFSE^low^ cells after co-culture with MDDC exposed to different virus minus the percentage of (mock-treated) CFSE^low^ cells.

### Cytokine and chemokine secretion by autologous MDDC-T cells co-cultures

The secretion of cytokines and chemokines induced during autologous MDDC-T cells co-cultures was measured in the culture supernatant using the Luminex technology (Cytokine Human 25-Plex Planel, Invitrogen), following the manufacturer’s instructions. The following 25 mediators were tested: Eotaxin, GM-CSF, IL-1β, IL-1RA, IL-2, IL-2R, IL-4, IL-5, IL-6, IL-7, IL-8, IL-10, IL-12p40/p70, IL-13, IL-15, IL-17, IFN-α, IFN-γ, IP-10, MCP-1, MIG, MIP-1α, MIP-1β, RANTES and TNF-α. Data were analyzed using the GraphPad Prism Software version 5.00 (San Diego, CA, USA). Comparisons for the different parameters were performed by Student’s test. For the analysis a level of significance was set at p < 0.05.

## Results

### Biochemical and antiretroviral activity of MUT-A

We previously reported the identification of an INLAI, Mut101 of the aryl or heteroaryl-tertbutoxy-acetic acid family [12]. In an effort to diversify our INLAI library and to obtain compounds with more potent ARV activity, we produced a library of 58 original INLAI compounds. These compounds share a common motif composed of a tert-butylether and a carboxylic acid group but linked to a heterocyclic scaffold different from those previously reported in this compound family. As shown in Fig. 1a, MUT-A is an original INLAI compound of 413 g/mol molecular weight, with a 5-membered heterocyclic thiophene core substituted by a pyridine group, a cyclohexen moiety and the sterically bulky tertbutoxy-ether key side chain. MUT-A has potent anti-HIV activity with an EC_50_ of 32 ± 9 nM or 12 ± 6 nM in MT4 cells infected by NL4-3 or HxB2 HIV-1 strains, respectively (Fig. 1b). As expected for all inhibitors of the INLAI family, MUT-A had a much weaker early ARV activity at integration, with an EC_50_ of 2.3 μM, measured in single-cycle infection of MT4 cells by NL4-3/Env VSVg-pseudotyped non-replicative virus (not shown). Cellular toxicity of MUT-A was low with CC_50_ of 42 ± 9 µM in MT4 cells, yielding a selectivity index of 1355. MUT-A inhibited IN (NL4-3)-LEDGF/p75 interaction with an IC_50_ of 92 ± 5 nM and promoted IN multimerization with an activation constant AC_50_ of 55 ± 2 nM and a maximum multimerization signal plateau of 600% compared to the background IN–IN signal obtained in the absence of MUT-A (Fig. 1c). The ARV activity of MUT-A was compared to that of previously described INLAIs (BI-D racemate and BI-224436) or to that of the second generation INSTI, Dolutegravir (DTG). MUT-A was found 4–6 times more potent than racemic BI-D and 1.4–1.7 times more potent than BI-224436, but 6–12 times less potent than DTG (Fig. 1d). Structure activity relationship of all compounds in this series demonstrates on the one hand, a tight correlation between IN-LEDGF/p75 inhibition and IN multimerization (Fig. 1e), and on the other hand, a good correlation between ARV activity and IN-LEDGF/p75 inhibition (Fig. 1f) or IN multimerization (Fig. 1g). As expected the correlation between ARV activity and in vitro biochemical activities on IN-LEDGF/p75 inhibition or IN multimerization was less tight than between the two different biochemical activities. Indeed, a parameter such as cellular permeability is critical for ARV activity but not relevant for the biochemical activities of these molecules. Altogether these results show that the MUT-A series are genuine INLAI compounds.

### Characterization of HIV-1 virions produced in the presence of MUT-A

We infected SupT1 T cells with the HIV-1 LAI strain and cultured the cells with or without MUT-A (160 nM, 5× EC_50_). In this experiment we produced in parallel HIV-1 LAI treated with another reference INLAI compound, BI-D, together with a control of virus produced in the absence of any compound that was previously published in [23]. Viral spread was monitored by measuring the CA-p24 level in the culture supernatant. Whereas efficient virus replication resulted in a rapid increase in CA-p24 level in the control culture, HIV-1 LAI was efficiently blocked by MUT-A (Additional file 1: Fig. S2A) similar to the antiviral effect of BI-D [23].

To test the effect of MUT-A on virus production, we transfected HEK 293T cells with the HIV-1 encoding plasmid pLAI [39] and cultured the cells in the presence or absence of MUT-A (Additional file 1: Fig. S2B) or BI-D [23]. The virus-containing supernatant was harvested 48 h later and the CA-p24 level was determined by ELISA. As previously shown for BI-D treatment, virus production on HEK 293T cells was not affected by MUT-A (Additional file 1: Fig. S2C). Western blot analysis of virions showed a similar protein content as untreated virions, while virions treated with Saquinavir (SQV) were clearly defective for protein maturation (Additional file 1: Fig. S2D). Next, we tested whether virus produced in the presence of MUT-A can promote a spreading infection in SupT1 T cells. No additional MUT-A was added during SupT1 culturing. Although we did not wash away MUT-A that is present in the virus stock, the high dilution factor (~ 2500×) and frequent passaging of the cell cultures (every 3–4 days), makes a sustained antiviral effect unlikely. Efficient viral spreading was observed upon infection of the cells with virus produced in the absence of MUT-A. In contrast, the presence of MUT-A or of BI-D during virus production severely impaired virus infectivity such that no spreading infection could be established (Additional file 1: Fig. S2E) as previously shown [23]. Similar results were obtained for the NL4-3 strain produced in the presence of MUT-A (data not shown). These effects have been reported for other INLAIs as well [7, 8, 12–14]. As previously described for BI-D [23], MUT-A treatment does not affect HIV-1 RNA packaging thermal stability of RNA dimers, reverse transcriptase activity and initiation of reverse transcription in vitro by tRNA^lys3^ primer (Additional file 1: Fig. S3).

One documented effect of INLAIs at the late phase of HIV-1 virion assembly and maturation is mislocalization of the ribonucleoprotein outside of the capsid core [9, 12, 14, 15]. We performed cryo-electron microscopy (cryo-EM) analysis on virus that were exposed or not to MUT-A treatment during virus production by 293T cells transfected with pNL4-3. The aim was to determine whether MUT-A, as previously described for other INLAIs [9, 15, 16], induced any architectural changes to the virus particle. Figure S4 illustrates the morphology of intact virus particles observed by cryo-EM, produced in the presence of MUT-A (Additional file 1: Fig. S4A) or its absence (Additional file 1: Fig. S4B). Variation in virus particle size can be observed in both panels, which is rather typical for HIV [40]. Particles were distinguished into three distinct phenotypes: (a) normal mature conical cores found mostly in virus produced in the absence of MUT-A (Additional file 1: Fig. S4B, blue arrow), (b) non-conical cores found both in virus produced in the presence of MUT-A (Additional file 1: Fig. S4A) and less frequently in the absence of MUT-A (Additional file 1: Fig. S4B), (c), virions with eccentric condensate, frequently found in virus produced in the presence of MUT-A (Additional file 1: Fig. S4A) and only very rarely observed for untreated virus. As previously indicated, these eccentric condensates likely represent the viral ribonucleoprotein (RNP) complex mislocalized outside the empty core. Indeed, these cryo-EM observations confirm that MUT-A treatment during virus production induces core abnormalities similar to those previously described for other integrase allosteric inhibitors, demonstrating that MUT-A displays all the characteristics attributed to allosteric inhibitors of integrase, such as IN multimerization, inhibition of IN-LEDGF/p75 interaction and induction of virion abnormalities such as cores with eccentric condensates.

### Resistance profile and spectrum of activity of MUT-A

**Fig. 2:**
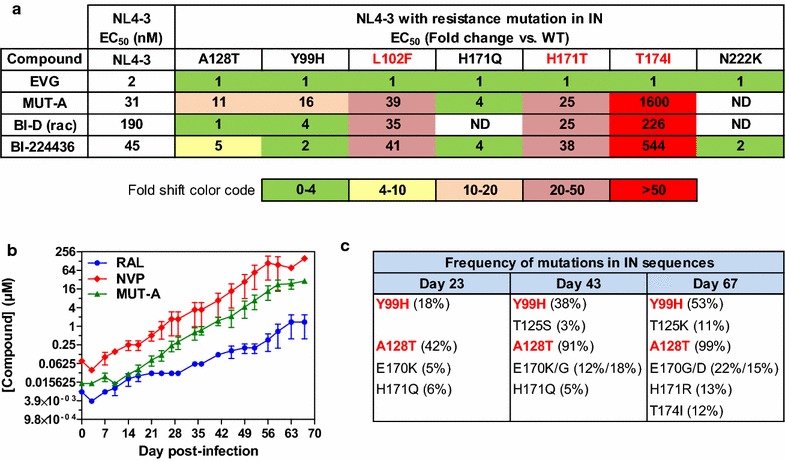
Resistance profile of MUT-A. **a** Comparative resistance profile of MUT-A and references (EVG, BI-D and BI-224436). The color code subdivides the fold change in resistance into five levels of magnitude. **b** Long-term culture of infected MT4 cells with escalating concentrations of drugs. Cells were initially infected at 0.005 MOI with HIV-1 and were passaged twice weekly in the presence of RAL (filled circle), NVP (filled diamond), or MUT-A (filled upward triangle). The serial passage was carried out over 67 days. The highest concentration of compounds at which the virus could replicate in culture are shown. Error bars represent the variation obtained in one experiment done in triplicate. **c** Frequency of mutations in IN sequences. Cloned sequences were analyzed at day 23, 43 and 67 from 30 to 40 individual clones (% of total clones analyzed)

Several mutations that induce resistance to INLAIs have been identified [6–13]. Since MUT-A is a new INLAI with an original scaffold never described previously, we wanted to check whether its potent ARV activity was sensitive to INLAI resistant mutations. First we constructed NL4-3 HIV-1 harboring IN single site mutations such as Y99H, A128T, L102F, H171Q, H171T, T174I, N222K, and checked the ARV activity of MUT-A and other INLAIs used as controls, BI-D and BI-224436, on each of these mutants. As shown on Fig. 2a, MUT-A unfortunately is impacted by all resistant mutants we tested, although at variable levels. The impact of these mutations was evaluated by their fold change in EC_50_. The most detrimental mutation is T174I with a considerable effect with a fold shift in EC_50_ as high as 1600. Then, mutations L102F, H171T, Y99H, A128T had a significant impact with fold changes of 39, 25, 16 and 11 respectively. Mutation H171Q had a lower impact with a fold change of 4. The other INLAIs used as controls, BI-D and BI-224436 had a similar profile of resistance with the T174I mutation having the strongest impact (EC_50_ fold change of 226 and 544 respectively) followed by L102F, H171T, Y99H or A128T. All these mutations are in the catalytic core domain (CCD) of IN, close to the INLAI binding site. On the contrary, mutation N222K is localized in the C-terminal domain (CTD) of IN at a distance of the binding site. Its impact is generally lower than the other mutations (data not shown for MUT-A, but see the effect on BI224436 in Fig. 2).

In addition to these experiments based on the fold change determination of MUT-A EC_50_ on purified mutant viruses, we sought to identify which resistant mutations are selected in serial passage experiment by progressively increasing MUT-A concentrations on MT4 cells infected with HIV-1 NL4-3, as described in the material and method section. Infected cells were passaged, starting at concentration below MUT-A EC_50_ (16 nM = EC_50_/2) to favor the occurrence of resistant mutations, and ending after 67 days selection at a MUT-A concentration 1000 fold higher (16 µM). Kinetics of MUT-A resistance was compared to resistance to Nevirapine (NVP, NRTI) and Raltegravir (RAL, INSTI). As shown in Fig. 2a, the kinetics of resistance development to MUT-A was parallel to that of NVP and slightly faster than that of RAL. At three different passages, day 23, day 43 and day 67, IN sequence analysis was performed by PCR in order to identify the resistant mutations that were selected and their frequency and kinetics of occurrence. As shown in Fig. 2b, c, the most abundant mutations that were detected as soon as day 23 are A128T and Y99H. These mutations remain the most frequent and accumulate with other minor mutations up to the end of the experiment. At day 67, A128T occurred in almost all sequences (99%) and Y99H in 53% of the sequences. Interestingly the most detrimental mutation, T174I, occurred only at the end with a frequency as low as 12%. The other strong resistant mutations, L102F and H171T were not detected at any time point. Some mutations such as H171Q which has a moderate impact (EC_50_ fold change of 4) were detected early at day 23 and then at day 43, but were not detectable at the end of the experiment.

These kinetics and frequency of occurrence of resistant mutations during serial passage experiment indicate that some mutations like A128T or Y99H arise preferentially compared to more detrimental mutations like T174I, L102F and H171T. One possible explanation of such phenomenon is that the resistance mutations that are favored correspond to virus resistant mutants that have the better replication capacity. We verified that this was indeed the case. As shown in Additional file 1: Fig. S5, viruses harboring Y99H or A128T resistance mutations have replication capacity similar or close to wt NL4-3, while viruses harboring H171T, T174I or L102F mutations had lower replication capacity. Presumably, during the resistance selection process, mutations that are favored are those which preserve the best virus fitness.

**Fig. 3:**
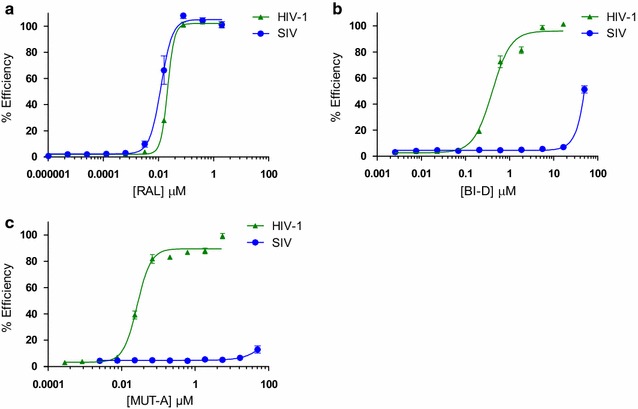
Antiviral activity in infection assays with HIV-1 and SIV. Dose–response curves for RAL (**a**), BI-D (**b**) and MUT-A (**c**) activity in MT4 cells infected with either HIV-1 NL4-3 (filled upward triangle) or SIVmac239 (filled circle). Error bars represent the variation obtained from three independent experiments done in duplicate

We then wanted to know if MUT-A was able or not to inhibit replication of other lentiviruses such as SIV. We compared the ARV activity of MUT-A, BI-D to that of the IN strand transfer inhibitor RAL used as control, on SIVmac239 and HIV-1 NL4-3. As shown in Fig. 3, while RAL had comparable efficiency on HIV-1 and SIVmac239 (reproducibly slightly better on SIV), neither MUT-A, nor BI-D had significant activity on SIV despite their potent activity on HIV-1. Similarly, MUT-A and BI-D had no significant activity on HIV-2 (data not shown).

### Immunoreactivity of MUT-A-inactivated HIV-1 against HIV-1 Env specific antibodies

**Fig. 4:**
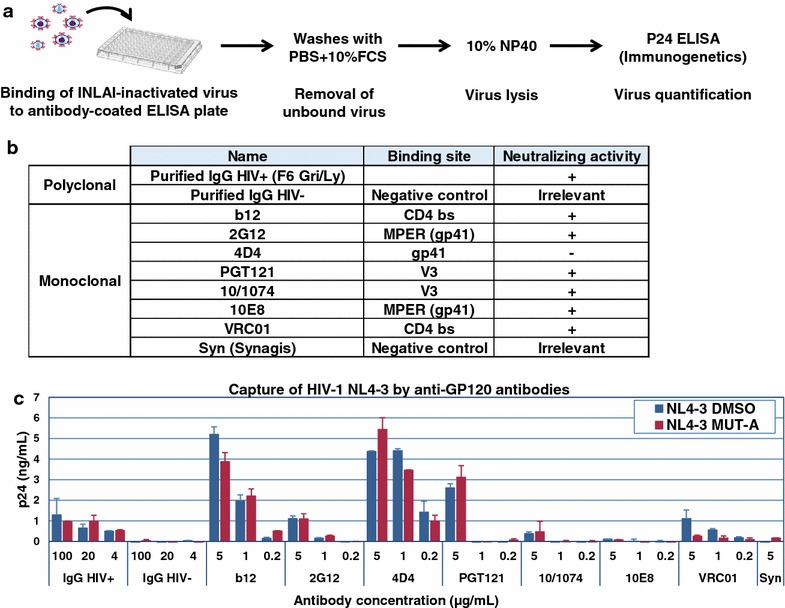
Immunoreactivity of HIV-1 produced in the presence or absence of MUT-A. **a** Schematic of the antibody capture assay of native HIV-1 particles produced. The capacity of the different antibodies to capture native HIV-1 particles was assessed by ELISA. HIV-1 particles retained by the antibodies were lysed and quantified by CA-p24 detection by ELISA. **b** The different monoclonal and polyclonal antibodies used in virus-capture assays are listed together with their specific target and their ability to neutralize HIV-1. Nonspecific antibodies (polyclonal IgG HIV- and mAb Syn = Synagis) were used as negative controls. **c** Quantitation by CA-p24 ELISA of native HIV-1 particles produced in the presence (red bars) or absence (blue bars) of MUT-A that have been captured with the different antibodies used at three different concentrations

We analyzed HIV-1 NL4-3 virions produced in the absence or presence of MUT-A for their immunoreactivity against a panel of polyclonal and monoclonal neutralizing and non-neutralizing anti-HIV-1 Env antibodies. Viral particles were captured by the antibodies coated on a 96-well ELISA plate and, after removal of unbound virus, we quantitated captured virus by detection of CA-p24 by ELISA after virus lysis (Fig. 4a). This assay allows the detection of capture of complete well-preserved virus particles [36]. The neutralizing and non-neutralizing antibodies used in these virus capture assays are listed in Fig. 4b together with their binding sites. This panel comprises two polyclonal antibodies: one HIV-1 specific neutralizing antibody (F6 Gri/Ly) and one non-specific IgG antibody as negative control, and seven anti-HIV Env monoclonal antibodies (mAbs) including two neutralizing mAbs directed against the CD4 binding site (b12 and VRC01), two neutralizing mAbs against the MPER gp41 epitope (2G12 and 10E8), a non-neutralizing mAb against gp41 (4D4), two neutralizing mAbs against the V3 Envelope epitope (PGT121 and 10/1074), and one irrelevant non-specific mAb (Synagis) as an additional negative control. Three antibody concentrations were used for each capture assay. As shown in Fig. 4c, NL4-3 MUT-A-inactivated particles were captured by the HIV Env specific antibodies with comparable efficiency as the non-treated NL4-3 particles. As expected, the two non-specific antibodies were negative in these capture assays. Except for the 10E8 and the 10/1074 mAbs which captured a comparable low amount of non-treated or inactivated virus, all specific antibodies were quite efficient in these virus capture assays. These results indicate that the MUT-A-inactivated NL4-3 virus particles were similarly immunoreactive as non-treated virus, indicating the preservation of the Envelope glycoprotein structure following inactivation of the virus particles. We next compared the immunoreactivity of HIV-1 virions produced in the presence of MUT-A with HIV-1 virions inactivated by other means such as AT-2 or Saquinavir treatment. As shown in Additional file 1: Table S1, Fig. S6 the immunoreactivities of these inactivated virions were comparable and similar to untreated virions.

### T-cell immunoreactivity of DCs from HIV^+^ patients loaded with MUT-A-inactivated HIV-1 NL4-3 co-cultured with autologous T lymphocytes

**Fig. 5:**
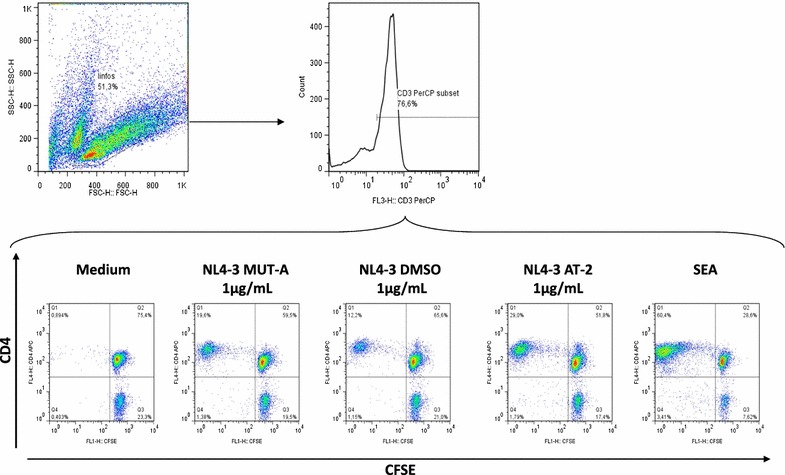
T-cell proliferation induced by autologous MDDCs exposed to MUT-A-inactivated HIV-1 assessed by CFSE proliferation assay. Proliferation in response to autologous MDDCs exposed to wt HIV-1 (NL4-3 DMSO) or MUT-A or AT-2 inactivated HIV-1 in a 6-day co-culture was assessed in triplicates using the CFSE proliferation assay. MDDCs were pulsed with virus particles corresponding to 1 μg/mL of HIV-1 Gag CA-p24. A representative (out of 3 independent experiments) flow cytometry dot plots showing lymphocyte gating strategy by FSC/SSC and CD3^+^ positive staining, as well as dot plots showing CFSE dilution in gated CD3^+^ CD4^+^ T lymphocytes after in vitro stimulation with the above mentioned virus or negative (medium) and positive SEA controls

**Fig. 6:**
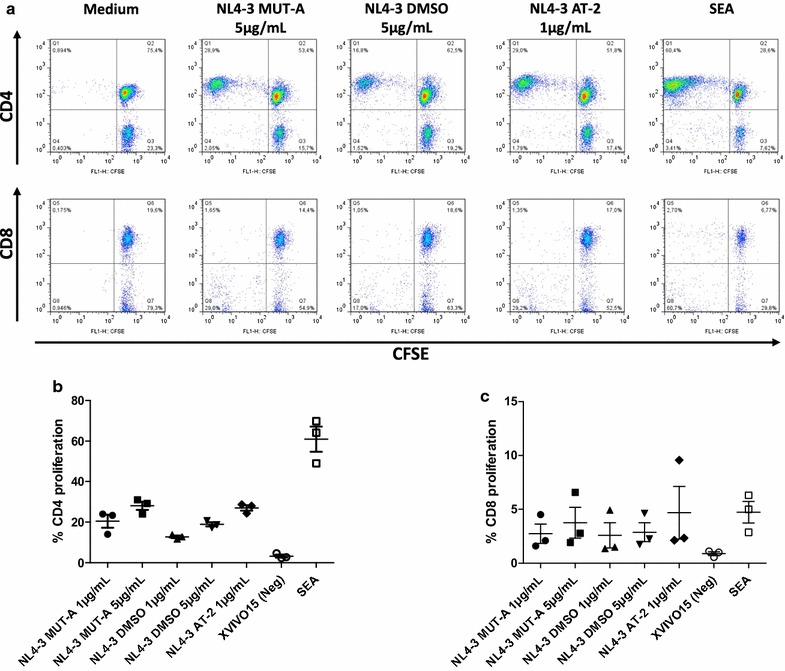
CD4^+^ and CD8^+^ T-cell proliferation induced by autologous MDDCs exposed to MUT-A-inactivated HIV-1. **a** Proliferation in response to autologous MDDC exposed to wt HIV-1 (NL4-3 DMSO) or MUT-A inactivated HIV-1 or AT-2-inactivated HIV-1 in a 6-day co-culture was assessed in triplicates using the CFSE proliferation assay. MDDCs were pulsed with either 5 μg/mL HIV-1 (NL4-3 DMSO or MUT-A) or 1 μg/mL HIV-1 (NL4-3 AT-2) of HIV-1 gag CA-p24. A representative (out of three independent experiments) flow cytometry dot plots showing CFSE dilution in gated CD3^+^ CD4^+^ and CD3^+^ CD8^+^ T lymphocytes after in vitro stimulation with the above mentioned viruses or negative (medium) and positive SEA controls. **b**, **c** Quantitation of T CD4^+^ (B) and CD8^+^ (C) proliferation in response to autologous MDDC exposed to wt HIV virus (NL4-3 DMSO) or inactivated HIV virus (MUT-A or NL4-3 AT-2) in a 6-day co-culture was assessed in triplicates using the CFSE proliferation assay. In all viruses used, MDDCs were pulsed with 1 µg/mL of HIV gag CA-p24. Graph showing mean ± SD of percentages of CFSE low CD3^+^ CD4^+^ (**b**) and CD3^+^ CD8^+^ (**c**) T cells from three different co-cultures are represented

We next investigated the T-cell immune responses induced by MDDC pulsed with non-treated and MUT-A-inactivated HIV-1 NL4-3, as well as AT-2-inactivated NL4-3 and the SEA super antigen as positive controls. After 6-days of co-culture, T cell proliferation induced by virus-loaded MDDC or MDCC pulsed with the SEA super antigen was estimated by loss of CFSE labelling (Fig. 5). Proliferation was observed essentially on CD4^+^ T cells (Fig. 5). Interestingly, the extend of CD4^+^ T lymphocyte proliferation observed with MDDCs loaded with MUT-A-inactivated NL4-3 was comparable or even higher than that obtained with MDDCs loaded with non-treated NL4-3 as shown in flow cytometry dot plots in Fig. 6a (28.9% loss of CFSE labelling for MUT-A inactivated NL4-3 versus 16.8% for non-treated NL4-3) or from quantitation of three independent experiments (Fig. 6b). This CD4^+^ T cell proliferation observed with MDDCs loaded with MUT-A-inactivated NL4-3 was also comparable to that obtained with AT-2-inactivated virus (29% loss in Fig. 6a), although smaller amount of AT-2-inactivated virion was used to obtain a similar level of CD4 proliferation. We also observed a small loss of CFSE labelling in CD8^+^ T cells. However, it seemed that CD8^+^ T lymphocyte reactivity was poor under the used experimental conditions, as shown by the weak loss of CFSE labelling obtained also with CD8 + T cells using MDDCs loaded with the super antigen SEA (2.7% as shown in Fig. 6a or about 5% on average in three independent experiments (Fig. 6c)).

**Fig. 7:**
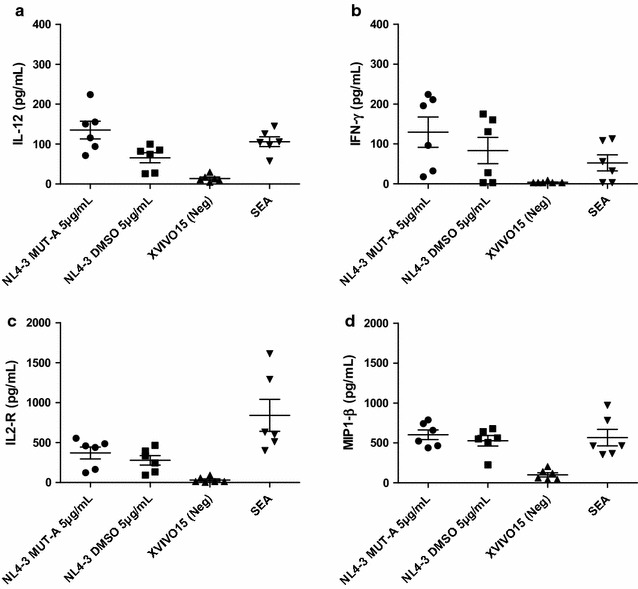
Secretion of IL-12, IFN-γ, IL2-R and MIP-1β induced by autologous MDDCs exposed to MUT-A-inactivated HIV-1. Cytokine and chemokine secretion in response to autologous MDDCs exposed to wt HIV-1 (NL4-3 DMSO) or MUT-A-inactivated HIV-1 (MUT-A 1 µM) in supernatants from a 6-day co-culture was assessed in duplicates by Luminex assay. Graph representing mean ± SD concentrations (pg/mL) of IL-12 (**a**), IFN-γ (**b**), IL2-R (**c**), and MIP-1β (**d**) analyzed after three different co-cultures

**Fig. 8:**
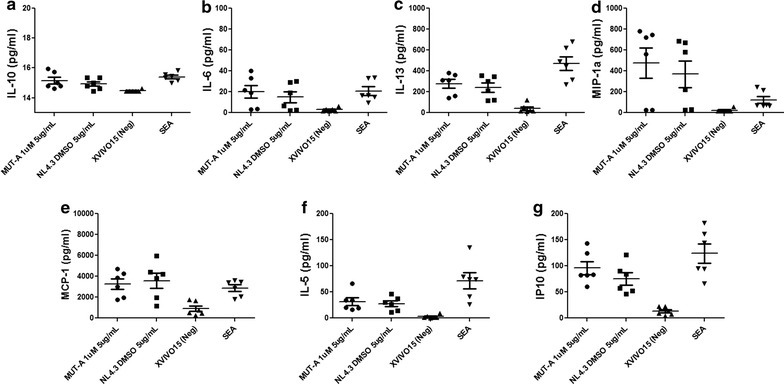
IL-5, IL-6, IL-10, IL-13, MIP-1α, MCP-1 and IP-10 secretion induced by MUT-A-HIV-1-exposed autologous MDDCs. Cytokine and chemokine secretion in response to autologous MDDCs exposed to wt HIV-1 (NL4-3 DMSO) or MUT-A-inactivated HIV-1 (MUT-A 1 µM) in supernatants from a 6-day co-culture was assessed in duplicates by Luminex assay. Graph representing mean ± SD concentrations (pg/mL) of IL-10 (**a**), IL-6 (**b**), IL-13 (**c**), MIP-1α (**d**), MCP-1 (**e**), IL-5 (**f**) and IP-10 (**g**) analyzed after three different co-cultures

To further characterize the effects of the MDDC exposure on MUT-A-inactivated-HIV-1 virus on the T-cell induced immune responses, the levels of secreted cytokines and chemokines in the culture supernatants of autologous pulsed MDDC and T cell co-cultures were assessed by Luminex technology. Results shown in Figs. 7 and 8 indicated that MDDC pulsed with MUT-A-inactivated NL4-3 induced secretion of IL-12 pro-inflammatory cytokine, Th1 cytokines (IFN-γ and IL-2R) as well as chemoattractant and antiviral chemokine (MIP-1β) (Fig. 7a–d), IL-10, IL-6, IL-13, MIP-1α, MCP-1, IL-5 and IP10 (IFN-γ-inducible protein 10). This pattern of cytokine/chemokine secretion reinforces the results obtained with CD4^+^ T cells proliferation assays and illustrates the significant induction of HIV-specific T cell immune response promoted by MUT-A-inactivated HIV-1 loaded on DCs isolated from HIV-1 infected subjects.

## Discussion

Here, we describe MUT-A as a new type of IN-LEDGF allosteric inhibitor. This compound consists of a 5-atom thiophene scaffold linked to the common carboxylic acid and tert-butylether moieties present on all INLAIs reported to date. As illustrated in Fig. 1, MUT-A as well as other compounds of the MUT-A series displayed biochemical activities specific of genuine INLAIs, such as inhibition of IN-LEDGF/p75 interaction and activation of IN multimerization. Furthermore, these two activities were tightly correlated, and both correlated with the antiretroviral activity of the MUT-A compounds (Fig. 1d–f). INLAIs that disrupt the IN-LEDGF/p75 interaction do not only affect the ‘early’ process of HIV-1 integration but, unexpectedly, also the ‘late’ process of virus maturation, triggering a strong infectivity defect when HIV-1 is produced in the presence of these inhibitors. This infectivity defect does not correlate with any detectable change in the protein content of these non-infectious virions and the maturation state of the structural Gag and Gag-Pol proteins as indicated by western blot analysis of MUT-A inactivated virus (Additional file 1: Fig. S2D). However, the MUT-A-induced infectivity defect correlated with the appearance of numerous capsid abnormalities in virus produced in the presence of MUT-A, such as virions that contain eccentric condensates outside of empty capsids as previously described [9, 15, 16], reconfirming that MUT-A is a genuine INLAI compound. We found that MUT-A, like other INLAIs, renders the progeny virus non-infectious when present during virus production. However, like the previous report on BI-D-treated viruses [23], we did not observe any effect on HIV-1 genomic RNA packaging thermal stability of RNA dimers, tRNA^lys3^ placement on the viral RNA and activity of the co-packaged RT enzyme. These results confirm and generalize the finding that the viral RNA genome and the initiation of reverse transcription is not a target of INLAIs. These combined results imply that RNA packaging and dimerization are not affected by the aberrant localization outside the virus core in INLAI-treated virions (as visible in the cryo-EM images, Additional file 1: Fig. S4), and suggest that RNA dimer maturation does not require a correct virus core conformation. Cell entry is not affected for virus produced in the presence of INLAIs, but there is a reverse transcription block in these target cells [9, 14, 15]. We show that the packaged RT enzyme is fully active on exogenous templates and that the tRNA^lys3^ primer is correctly placed on the genomic RNA for cDNA synthesis. Thus, all basic factors required for reverse transcription are functionally present in INLAI-treated virions, which nevertheless are severely defective in reverse transcription. Several studies indicated that the first step of reverse transcription (initiation and production of strong-stop cDNA) is inhibited [9, 14, 15]. This may indicate that the components, although present and active in INLAI-treated virions, are not located at the right position at the right time to execute the complex reverse transcription reaction.

Although MUT-A harbors an original 5-membered thiophene scaffold, it is nevertheless as sensitive as the other INLAIs to several point resistance mutations in the IN gene. These mutations are mainly located close to the INLAI binding site in the CCD, with the exception of the N222K mutation localized in the CTD of IN. Presumably mutations in the CCD could strongly diminish the binding affinity of MUT-A and other INLAIs for IN, disrupting their interaction with IN and consequently decrease strongly or cancel their ARV activity. The impact of these mutations on compound binding is due to impaired interaction with the conserved common carboxylic acid and tert-butylether moieties that are found in all INLAIs rather than with the scaffold moiety.

On the contrary, the N222K resistant mutation that is localized in the IN CTD, at a distance from the compound binding site, could impact the conformational change induced by INLAIs for IN multimerization rather than the compound binding affinity. The resistance profile of MUT-A, BI-D and BI-224436 indicate that the genetic barrier of these compounds is weak, similar to that of NVP, since they are very sensitive to point resistant mutants. Fortunately, on the one hand, INSTIs like Elvitegravir (EVG) have fully conserved activity on these mutants, and on the other hand INLAIs conserved full ARV activity on mutants resistant to INSTIs [12]. These results allow us to consider a future combination of INLAIs and INSTIs as fully efficient. The lack of activity of INLAIs on SIV or HIV-2 compared to the fully conserved activity of INSTIs on HIV-1, HIV-2 and SIV is reminiscent of the inefficiency of NNRTIs on SIV and HIV-2. This seems to be a general property of allosteric inhibitors in contrast with catalytic inhibitors of both IN and RT enzymes.

Since we could not observe any detectable change in the protein content of MUT-A-inactivated HIV-1 neither in the packaging of viral RNA nor in exogenous RT activity, we completed this analysis by inspecting further the immunoreactivity of these MUT-A-inactivated HIV-1 particles. We indeed found that these inactivated viruses exhibit conserved B and T cell immunoreactivity, comparable with that of non-treated HIV-1. MUT-A-inactivated viruses were captured by multiple neutralizing and non-neutralizing polyclonal and monoclonal anti-HIV Env antibodies with efficiency comparable to that of non-treated HIV-1. These results indicate that MUT-A-inactivated HIV-1 particles conserve various important native epitopes for a B-cell immune response such as the CD4 binding site and V3 epitope on the gp120 Env surface subunit or the major proximal epitope of the gp41 trans-membrane subunit. We also found that MUT-A-inactivated virus had B-cell immunoreactivity toward this panel of antibodies comparable to that of HIV-1 NL4-3 inactivated by other means such as Protease inhibitor or AT-2 treatment (Additional file 1: Fig. S6).

In addition to the exploration of the immunoreactivity of MUT-A-inactivated HIV-1 with regard to this panel of neutralizing and non-neutralizing anti-Env antibodies, we studied if these inactivated viruses were suitable to elicit a CD4^+^ and CD8^+^ T-cells immune response when loaded ex vivo on MDDCs isolated from subjects infected by HIV-1. We measured a strong CD4^+^ T helper cell proliferative response, comparable or even higher than that induced by non-treated viruses. This indicates that MUT-A-inactivated viruses are well recognized by professional antigen presenting cells. In contrast, the CD8^+^ T cell proliferative response was much weaker, but the meaning of this was not obvious since we also observed a very weak CD8 response against the SAE super antigen that was used as positive control.

These results suggest that MUT-A treatment during virus production did not alter the conformation of epitopes at the surface of the virus. In fact, previous work has indicated that, for MDDC, small amounts of antigen are sufficient for presentation on MHC molecules and activation of T cells. Some of these approaches have been used with antigens from non-replicating viruses, including aldrithiol-2 (AT-2)-inactivated HIV-1 [41–43] or defective virus [44]. As we describe in this report, MUT-A-inactivated virus conserves conformational and functionally intact surface envelope proteins that can interact with T cells as well as with surface receptors present on circulating dendritic cells and monocytes. One of the advantages of MUT-A inactivation over other modes of inactivation such as AT-2 treatment is that MUT-A is a non-toxic ARV compound in development compared to AT-2 which is a toxic and denaturing agent. The potential use of AT-2-treated biological would be certainly prohibited in human. Also, the well documented observation that INLAI-inactivated virus enters target cells as wt virus and could accumulate and be degraded before the reverse transcription block might also confer an advantage in term of cellular immune response in vivo. Compared to HIV-1 inactivation by protease inhibitors, INLAI-inactivated viruses did not display any Gag maturation defect (Additional file 1: Fig. S2). These combined results make INLAI-treated virus an interesting reagent that could be exploited for vaccine research in animal models of HIV infection. Unfortunately, the lack of ARV activity of INLAIs against SIV precludes the use of the macaque model infected with SIV. The only possible model that could be used today for such studies would be the humanized mouse model infected with HIV-1, although the immune B cell response is somehow limited in the current humanized mouse models.

## Conclusions

In conclusion, this report describes the characteristics of a new type of INLAI drug MUT-A, which consists of an original scaffold, a 5-atom thiophene ring, linked to carboxylic acid and tert-butylether moieties common to most INLAIs. We demonstrate that HIV-1 inactivated by MUT-A displays a strong defect in infectivity. However, this strong infectivity defect did not correlate with detectable change in the viral protein content, Gag maturation, viral RNA packaging and exogenous RT activity, but correlated with the formation of numerous viral particles containing eccentric condensates with empty cores. We described resistance mutations that strongly reduce the ARV activity of this class of compounds. In addition, from the point of view of both the B cell immunoreactivity against a panel of neutralizing and non-neutralizing anti-HIV-1 Env antibodies, as well as the T CD4^+^ helper cell proliferative response, the MUT-A-inactivated HIV-1 behaves like a strong and efficient antigen capable to promote large B and T cells immune responses comparable or even higher than non-treated virus. Up to date the only structural difference that has been observed between INLAI-inactivated HIV-1 and non-treated virus is the formation of an eccentric core and misplacement of the genetic RNA material outside the viral core. The work reported here concerning the B and T cell immunoreactivities displayed by MUT-A inactivated HIV-1 suggests that the surface of such inactivated virions, which plays an essential role in eliciting B and T cell immune responses, seems to be unaltered. However, all this work has been performed with two selected HIV-1 isolates, the primary X4-using LAI isolate and the X4-using lab-adapted NL4-3 virus. It remains to be seen whether these observations could be extended to multiple HIV-1 primary isolates. If this is the case, it would be worth checking whether the use of such INLAI-inactivated viruses as immunogen in some adapted animal models suffices to raise a protective neutralizing antibody response against HIV and could be used as therapeutic adjuvant to ART that could be tested in a small animal model of HIV-1 infection. Interestingly, while this manuscript was in preparation, Vranckx and colleagues [18] published a paper showing that LEDGINs can relocate and retarget HIV integration in a HIV reservoir that is refractory to reactivation by different latency-reversing agents, supporting the notion that this INLAI class of compounds could reduce reactivation of residual latent HIV. In a commentary on this work, Mesplede and Wainberg [45] raise the intriguing possibility that LEDGINs could play a role in a cure for HIV infection. The work presented here, together with that of Vranckx et al. [18] suggests that it is worthwhile to explore these novel possibilities and to test whether INLAIs could be exploited as adjuvant for HIV cure.

## Additional file

**Additional file 1.** Supplementary methods. Chemical synthesis process of MUT-A,HTRF^®^-based IN-LEDGF interaction assay, HTRF^®^-based IN multimerization assay, Viral RNA isolation, Northern blot analyses, primer extension assays, RT activity assays, Cryo-electron microscopy of HIV particles, Determination of HIV-1 replication capacity. **Figure S1:** 1H NMR spectrum of MUT-A. **Figure S2:** Impact of MUT-A on HIV-1 replication and production. **Figure S3:** Analysis of genomic RNA, Reverse transcriptase and tRNALys3 primer in MUT-A-treated HIV-1. A-D. HIV-1 RNA packaging and thermal stability of HIV-1 RNA dimers. **Figure S4:** HIV-1 NL4-3 particles observed by cryo-EM. A. **Figure S5:** Replicative capacity of HIV-1 NL4-3 viruses bearing resistance mutations to INLAIs or INSTIs. **Table S1:** Immunoreactivity of HIV-1 NL4-3 produced in the presence of MUT-A, or Saquinavir, or after AT2 treatment, or in the presence of DMSO. **Figure S6:** Results of virus immunocapture from Table S1 represented in bar graphs.

## References

[1] Lesbats P, Engelman AN, Cherepanov P (2016). Retroviral DNA integration. Chem Rev.

[2] Engelman A, Cherepanov P (2008). The lentiviral integrase binding protein LEDGF/p75 and HIV-1 replication. PLoS Pathog.

[3] Sowd GA, Serrao E, Wang H, Wang W, Fadel HJ, Poeschla EM (2016). A critical role for alternative polyadenylation factor CPSF6 in targeting HIV-1 integration to transcriptionally active chromatin. Proc Natl Acad Sci.

[4] Cherepanov P, Maertens G, Proost P, Devreese B, Jozef VB, Engelborghs Y (2003). HIV-1 integrase forms stable tetramers and associates with LEDGF/p75 protein in human cells. J Biol Chem.

[5] Emiliani S, Mousnier A, Busschots K, Maroun M, Van Maele B, Tempé D (2005). Integrase mutants defective for interaction with LEDGF/p75 are impaired in chromosome tethering and HIV-1 replication. J Biol Chem.

[6] Christ F, Voet A, Marchand A, Nicolet S, Desimmie BA, Marchand D (2010). Rational design of small-molecule inhibitors of the LEDGF/p75-integrase interaction and HIV replication. Nat Chem Biol.

[7] Kessl JJ, Jena N, Koh Y, Taskent-Sezgin H, Slaughter A, Feng L (2012). Multimode, cooperative mechanism of action of allosteric HIV-1 integrase inhibitors. J Biol Chem.

[8] Tsiang M, Jones GS, Niedziela-Majka A, Kan E, Lansdon EB, Huang W (2012). New class of HIV-1 integrase (IN) inhibitors with a dual mode of action. J Biol Chem.

[9] Balakrishnan M, Yant SR, Tsai L, O’Sullivan C, Bam RA, Tsai A (2013). Non-catalytic site HIV-1 integrase inhibitors disrupt core maturation and induce a reverse transcription block in target cells. PLoS ONE.

[10] Fenwick C, Amad M, Bailey MD, Bethell R, Bös M, Bonneau P (2014). Preclinical profile of BI 224436, a novel HIV-1 non-catalytic-site integrase inhibitor. Antimicrob Agents Chemother.

[11] Sharma A, Slaughter A, Jena N, Feng L, Kessl JJ, Fadel HJ (2014). A new class of multimerization selective inhibitors of HIV-1 integrase. PLoS Pathog.

[12] Le Rouzic E, Bonnard D, Chasset S, Bruneau J-M, Chevreuil F, Le Strat F (2013). Dual inhibition of HIV-1 replication by integrase-LEDGF allosteric inhibitors is predominant at the post-integration stage. Retrovirology.

[13] Christ F, Shaw S, Demeulemeester J, Desimmie BA, Marchand A, Butler S (2012). Small-molecule inhibitors of the LEDGF/p75 binding site of integrase block HIV replication and modulate integrase multimerization. Antimicrob Agents Chemother.

[14] Desimmie BA, Schrijvers R, Demeulemeester J, Borrenberghs D, Weydert C, Thys W (2013). LEDGINs inhibit late stage HIV-1 replication by modulating integrase multimerization in the virions. Retrovirology.

[15] Jurado KA, Wang H, Slaughter A, Feng L, Kessl JJ, Koh Y (2013). Allosteric integrase inhibitor potency is determined through the inhibition of HIV-1 particle maturation. Proc Natl Acad Sci USA.

[16] Fontana J, Jurado KA, Cheng N, Ly NL, Fuchs JR, Gorelick RJ (2015). Distribution and redistribution of HIV-1 nucleocapsid protein in immature, mature, and integrase-inhibited virions: a role for integrase in maturation. J Virol.

[17] Kessl JJ, Kutluay SB, Townsend D, Rebensburg S, Slaughter A, Larue RC (2016). HIV-1 integrase binds the viral RNA genome and is essential during virion morphogenesis. Cell.

[18] Vranckx LS, Demeulemeester J, Saleh S, Boll A, Vansant G, Schrijvers R (2016). LEDGIN-mediated inhibition of Integrase–LEDGF/p75 interaction reduces reactivation of residual latent HIV. EBioMedicine.

[19] Girard MP, Osmanov S, Assossou OM, Kieny M-P (2011). Human immunodeficiency virus (HIV) immunopathogenesis and vaccine development: a review. Vaccine.

[20] Garcia F, Routy J-P (2011). Challenges in dendritic cells-based therapeutic vaccination in HIV-1 infection. Vaccine.

[21] Garcia F, Climent N, Guardo AC, Gil C, Leon A, Autran B (2013). A dendritic cell-based vaccine elicits T cell responses associated with control of HIV-1 replication. Sci Transl Med.

[22] Chasset S, Chevreuil F, Ledoussal B, Le Strat F, Benarous R. Inhibitors of viral replication, their process of preparation and their therapeutical uses. WO2014/053666A1. 2014.

[23] van Bel N, van der Velden Y, Bonnard D, Le Rouzic E, Das AT, Benarous R (2014). The allosteric HIV-1 Integrase inhibitor BI-D affects virion maturation but does not influence packaging of a functional RNA genome. PLoS ONE.

[24] Rutebemberwa A, Bess JW, Brown B, Arroyo M, Eller M, Slike B (2007). Evaluation of aldrithiol-2-inactivated preparations of HIV type 1 subtypes A, B, and D as reagents to monitor T cell responses. AIDS Res Hum Retroviruses.

[25] Kodama T, Wooley DP, Naidu YM, Kestler HW, Daniel MD, Li Y (1989). Significance of premature stop codons in env of simian immunodeficiency virus. J Virol.

[26] Moog C, Dereuddre-Bosquet N, Teillaud J-L, Biedma ME, Holl V, Van Ham G (2014). Protective effect of vaginal application of neutralizing and nonneutralizing inhibitory antibodies against vaginal SHIV challenge in macaques. Mucosal Immunol.

[27] Wu X, Yang Z-Y, Li Y, Hogerkorp C-M, Schief WR, Seaman MS (2010). Rational design of envelope identifies broadly neutralizing human monoclonal antibodies to HIV-1. Science.

[28] Walker LM, Phogat SK, Chan-Hui P-Y, Wagner D, Phung P, Goss JL (2009). Broad and potent neutralizing antibodies from an African donor reveal a new HIV-1 vaccine target. Science.

[29] Zhou T, Xu L, Dey B, Hessell AJ, Van Ryk D, Xiang S-H (2007). Structural definition of a conserved neutralization epitope on HIV-1 gp120. Nature.

[30] Trkola A, Purtscher M, Muster T, Ballaun C, Buchacher A, Sullivan N (1996). Human monoclonal antibody 2G12 defines a distinctive neutralization epitope on the gp120 glycoprotein of human immunodeficiency virus type 1. J Virol.

[31] Muster T, Steindl F, Purtscher M, Trkola A, Klima A, Himmler G (1993). A conserved neutralizing epitope on gp41 of human immunodeficiency virus type 1. J Virol.

[32] Stiegler G, Kunert R, Purtscher M, Wolbank S, Voglauer R, Steindl F (2001). A potent cross-clade neutralizing human monoclonal antibody against a novel epitope on gp41 of human immunodeficiency virus type 1. AIDS Res Hum Retroviruses.

[33] Zwick MB, Labrijn AF, Wang M, Spenlehauer C, Saphire EO, Binley JM (2001). Broadly neutralizing antibodies targeted to the membrane-proximal external region of human immunodeficiency virus type 1 glycoprotein gp41. J Virol.

[34] Lorin V, Mouquet H (2015). Efficient generation of human IgA monoclonal antibodies. J Immunol Methods.

[35] Kwong PD, Mascola JR (2012). Human antibodies that neutralize HIV-1: identification, structures, and B cell ontogenies. Immunity.

[36] Burrer R, Haessig-Einius S, Aubertin A-M, Moog C (2005). Neutralizing as well as non-neutralizing polyclonal immunoglobulin (Ig)G from infected patients capture HIV-1 via antibodies directed against the principal immunodominant domain of gp41. Virology.

[37] Nyambi PN, Burda S, Bastiani L, Williams C (2001). A virus binding assay for studying the antigenic landscape on intact, native, primary human immunodeficiency virus-type 1. J Immunol Methods.

[38] Climent N, Guerra S, García F, Rovira C, Miralles L, Gómez CE (2011). Dendritic cells exposed to MVA-based HIV-1 vaccine induce highly functional HIV-1-specific CD8(+) T cell responses in HIV-1-infected individuals. PLoS ONE.

[39] Peden K, Emerman M, Montagnier L (1991). Changes in growth properties on passage in tissue culture of viruses derived from infectious molecular clones of HIV-1LAI, HIV-1MAL, and HIV-1ELI. Virology.

[40] Zhang W, Cao S, Martin JL, Mueller JD, Mansky LM (2015). Morphology and ultrastructure of retrovirus particles. AIMS Biophys.

[41] Bender A, Bui LK, Feldman MA, Larsson M, Bhardwaj N (1995). Inactivated influenza virus, when presented on dendritic cells, elicits human CD8^+^ cytolytic T cell responses. J Exp Med.

[42] Larsson M, Fonteneau J-F, Lirvall M, Haslett P, Lifson JD, Bhardwaj N (2002). Activation of HIV-1 specific CD4 and CD8 T cells by human dendritic cells: roles for cross-presentation and non-infectious HIV-1 virus. AIDS..

[43] Buseyne F, Le Gall S, Boccaccio C, Abastado JP, Lifson JD, Arthur LO (2001). MHC-I-restricted presentation of HIV-1 virion antigens without viral replication. Nat Med.

[44] Guardo AC, Alvarez-Fernández C, Arberas H, García-Pérez J, García F, Bargalló ME (2013). Use of RT-defective HIV virions: new tool to evaluate specific response in chronic asymptomatic HIV-infected individuals. PLoS ONE.

[45] Mesplède T, Wainberg MA (2016). Will LEDGIN molecules be able to play a role in a cure for HIV infection?. EBioMedicine.

