# Supplementary material for: The HIV-1 integrase-LEDGF allosteric inhibitor MUT-A: resistance profile, impairment of virus maturation and infectivity but without influence on RNA packaging or virus immunoreactivity
Source: Retrovirology. 2017 Nov 9;14:50. doi: 10.1186/s12977-017-0373-2 (PMC5680779; doi:10.1186/s12977-017-0373-2)

Additional File 1. Supplementary methods. Chemical synthesis process of MUT-A, HTRF®-based IN-LEDGF interaction assay, HTRF®-based IN multimerization assay, Viral RNA isolation, Northern blot analyses, primer extension assays, RT activity assays, Cryo-electron microscopy of HIV particles, Determination of HIV-1 replication capacity. **Figure S1:** 1H NMR spectrum of MUT-A. **Figure S2:** Impact of MUT-A on HIV-1 replication and production. **Figure S3:** Analysis of genomic RNA, Reverse transcriptase and tRNALys3primer in MUT-A-treated HIV-1. A-D. HIV-1 RNA packaging and thermal stability of HIV-1 RNA dimers. **Figure S4:** HIV-1 NL4-3 particles observed by cryo-EM. A. **Figure S5:** Replicative capacity of HIV-1 NL4-3 viruses bearing resistance mutations to INLAIs or INSTIs. **Table S1:** immunoreactivity of HIV-1 NL4-3 produced in the presence of MUT-A, or Saquinavir, or after AT2 treatment, or in the presence of DMSO. **Figure S6:** Results of virus immunocapture from Table S1 represented in bar graphs

Supplementary methods

Chemical synthesis process of MUT-A ((2S)-2-(*tert*-butoxy)-2-[4-(4,4-dimethylcyclohex-1-en-1-yl)-2-methyl-5-(pyridin-4-yl)thiophen-3-yl]acetic acid):

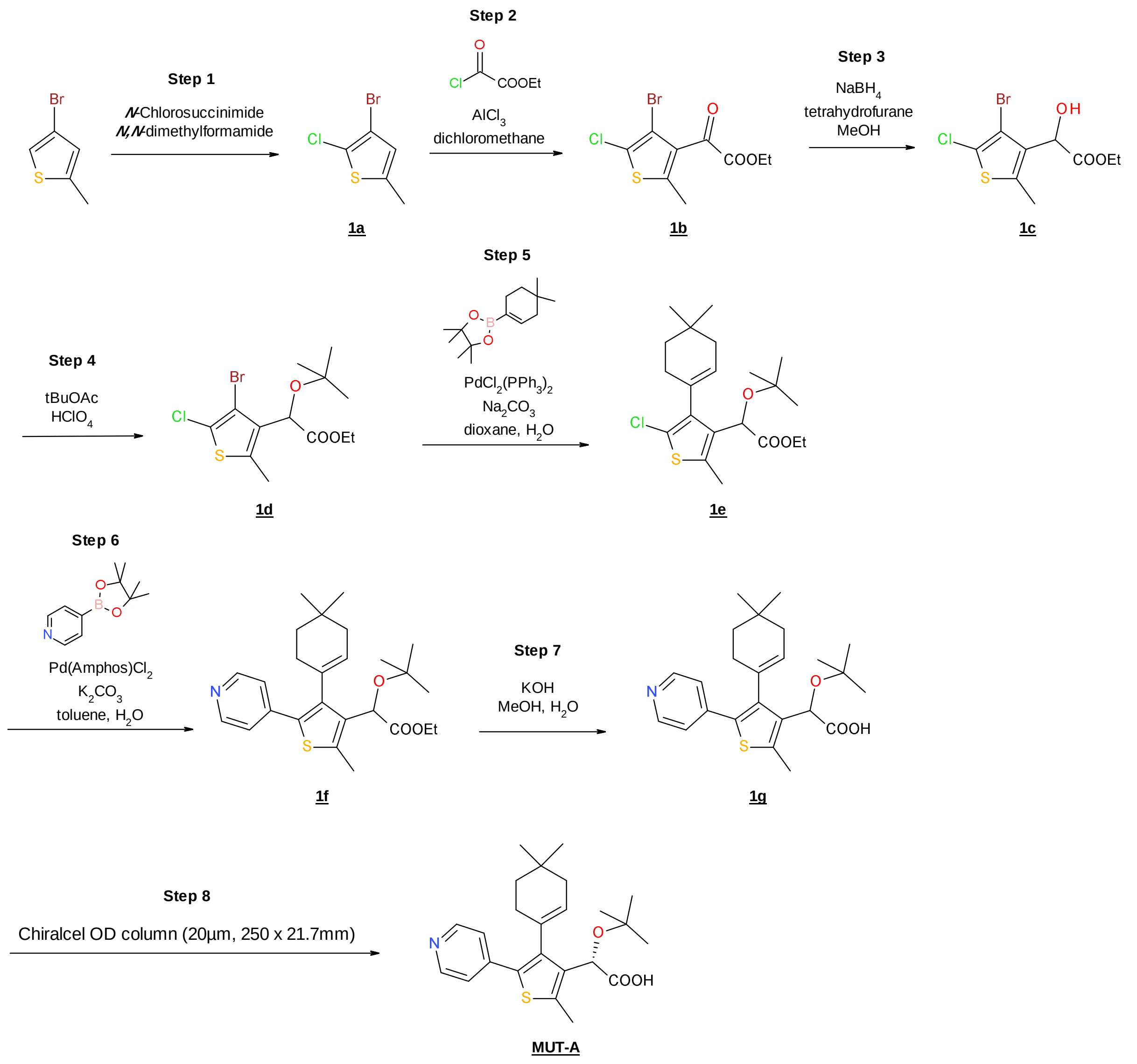

Step 1: preparation of intermediate 3-bromo-2-chloro-5-methylthiophene **(1a)**

Under argon atmosphere, *N*-chlorosuccinimide (1.51 g, 11.3 mmol) was added at -5°C per portion to a solution of 4-bromo-2-methylthiophene (2 g, 11.3 mmol) in *N,N*-dimethylformamide (6 mL) in an amber round bottom flask. After 1 hour at 0°C, the reaction mixture was warmed to room temperature and stirred for 3 hours more. Water was then added at 0°C and the mixture was extracted with dichloromethane twice. The organic layer was washed with brine, dried over sodium sulfate, filtered, and evaporated under reduced

pressure. The residue was purified by flash chromatography on silica gel (cyclohexane as the eluent) to afford the 3-bromo-2-chloro-5-methylthiophene **(1a)** as yellow oil (1.71 g, 8.1 mmol, 71%).

^{1}H NMR (400 MHz, $CDCl_3$) δ 2.40 (d, J = 1.1 Hz, 3H), 6.58 (d, J = 1.1 Hz, 1H).

<u>Step 2</u>: preparation of intermediate ethyl 2-(4-bromo-5-chloro-2-methylthiophen-3-yl)-2-oxoacetate **(1b)**

Under a nitrogen atmosphere, ethyl chlorooxoacetate (898 µL, 8.04 mmol) and aluminum chloride (III) (2.14 g, 16.07 mmol) were added successively at -10°C to a solution of 3-bromo-2-chloro-5-methylthiophene **(1a)** (1.7 g, 8.04 mmol) in dichloromethane (84 mL). After 1 hour at 0°C, the reaction mixture was stirred at room temperature for 8 hours and then slowly hydrolyzed at 0°C with water. The organic layer was separated, washed with an 1N hydrochloric acid aqueous solution, brine, dried over sodium sulfate, filtered and evaporated under reduced pressure. The residue was purified by flash chromatography on silica gel (cyclohexane/ethyl acetate 90/10) to give ethyl 2-(4-bromo-5-chloro-2-methylthiophen-3-yl)-2-oxoacetate **(1b)** as an orange solid (1.44 g, 4.6 mmol, 56%).

^{1}H NMR (400 MHz, $CDCl_3$) δ 1.41 (t, J = 7.2 Hz, 3H), 2.61 (s, 3H), 4.41 (q, J = 7.2 Hz, 2H).

<u>Step 3</u>: preparation of intermediate ethyl 2-(4-bromo-5-chloro-2-methylthiophen-3-yl)-2-hydroxyacetate **(1c)**

To a solution of ethyl 2-(4-bromo-5-chloro-2-methylthiophen-3-yl)-2-oxoacetate **(1b)** (340 mg, 1.09 mmol) in a mixture of tetrahydrofuran (7 mL) and ethanol (1.7 mL) was added sodium tetraborohydride (37.2 mg, 0.98 mmol) at 0°C. The mixture was stirred at 0°C for 2 hours. The mixture was quenched with a 1N hydrochloric acid aqueous solution and extracted with ethyl acetate twice. The organic extract was washed with brine twice, dried over sodium sulfate, filtered and concentrated to afford the crude ethyl 2-(4-bromo-5-chloro-2-methylthiophen-3-yl)-2-hydroxyacetate **(1c)** as a colorless oil (335 mg, 1.07 mmol, 98%).

^{1}H NMR (400 MHz, $CDCl_3$) δ 1.27 (t, J = 7.1 Hz, 3H), 2.42 (s, 3H), 4.23-4.32 (m, 2H), 5.27 (s, 1H).

<u>Step 4</u>: preparation of intermediate ethyl 2-(4-bromo-5-chloro-2-methylthiophen-3-yl)-2-(*tert*-butoxy)acetate **(1d)**

To a suspension of ethyl 2-(4-bromo-5-chloro-2-methylthiophen-3-yl)-2-hydroxyacetate **(1c)** (334 mg, 1.065 mmol) in *tert*-butylacetate (10.9 mL) at -5°C was added perchloric acid (70%, 0.4 mL). The reaction mixture was stirred at -5°C for 1 hour then at 0°C for 30 minutes more and at room temperature for 1hour more. The reaction mixture was basified with a saturated aqueous solution of sodium bicarbonate until pH 8. The aqueous layer was extracted with ethyl acetate. The organic layer was washed with brine, dried over sodium sulfate, filtered and concentrated *in vacuo*. The residue was purified by flash chromatography on silica gel (cyclohexane/ethyl acetate 95/5) to afford ethyl 2-(4-bromo-5-chloro-2-methylthiophen-3-yl)-2-(*tert*-butoxy)acetate **(1d)** as a colorless oil (324 mg, 0.88 mmol, 82 %).

^{1}H NMR (400 MHz, $CDCl_3$) δ 1.20-1.23 (m, 12H), 2.51 (s, 3H), 4.08-4.21 (m, 2H), 5.22 (s, 1H).

<u>Step 5</u>: preparation of intermediate ethyl 2-(*tert*-butoxy)-2-[5-chloro-4-(4,4-dimethylcyclohex-1-en-1-yl)-2-methylthiophen-3-yl]acetate **(1e)**

Under argon atmosphere, ethyl 2-(4-bromo-5-chloro-2-methylthiophen-3-yl)-2-(*tert*-butoxy)acetate **(1d)** (500 mg, 1.35 mmol), 4,4-(dimethylcyclohexene-1-yl)boronic acid pinacol ester (319.5 mg, 1.35 mmol), sodium carbonate (429 mg, 4.05 mmol) were dissolved in dioxane (28 mL) and water (4 mL). The solution was degassed under argon for 10 minutes and bis(triphenylphosphine)palladium (II) dichloride (142 mg, 0.20 mmol) was added. The reaction was heated and shaken at 85°C, for 8 hours. After cooling at room temperature, the mixture was filtered through celite®, rinsed with methanol. The filtrate was concentrated *in vacuo* and partitioned between ethyl acetate and water. The organic layer was washed with brine, dried over sodium sulfate, filtered and concentrated under reduced pressure. The

crude material was purified by flash chromatography on silica gel (cyclohexane/ethyl acetate 97/3) to give ethyl 2-(*tert*-butoxy)-2-[5-chloro-4-(4,4-dimethylcyclohex-1-en-1-yl)-2-methylthiophen-3-yl]acetate **(1e)** (268 mg, 0.67 mmol, 37%).

^{1}H NMR (400 MHz, $CDCl_3$) δ 1.02 (s, 3H), 1.03 (s, 3H), 1.17 (s, 9H), 1.21 (t, J = 7.1 Hz, 3H), 1.47-1.51 (m, 2H), 1.91-2.03 (m, 2H), 2.07-2.15 (m, 1H), 2.31-2.39 (m, 1H), 2.48 (s, 3H), 4.06-4.18 (m, 2H), 4.97 (s, 1H), 5.53-5.60 (bs, 1H).

Step 6: preparation of intermediate ethyl 2-(*tert*-butoxy)-2-[4-(4,4-dimethylcyclohex-1-en-1-yl)-2-methyl-5-(pyridin-4-yl)thiophen-3-yl]acetate **(1f)**

Under argon atmosphere, ethyl 2-(*tert*-butoxy)-2-[5-chloro-4-(4,4-dimethylcyclohex-1-en-1-yl)-2-methylthiophen-3-yl]acetate **(1e)** (100 mg, 0.251 mmol), 4-pyridine boronic acid pinacol ester (128.5 mg, 0.63 mmol), potassium carbonate (69.3 mg, 0.50 mmol) were dissolved in toluene (1.65 mL) and water (0.16 mL). The solution was degassed under argon for 10 minutes and bis(di-*tert*-butyl(4-dimethylaminophenyl)phosphine)dichloropalladium(II) (35.2 mg, 0.05 mmol) was added. The reaction was heated and shaken at 90°C for 18 hours. After cooling at room temperature, water was added and the mixture was extracted twice with ethyl acetate. The organic layer was washed with brine, dried over sodium sulfate, filtered, and concentrated under reduced pressure. The crude material was purified by preparative TLC (cyclohexane/ethyl acetate 70/30) to give ethyl 2-(*tert*-butoxy)-2-[4-(4,4-dimethylcyclohex-1-en-1-yl)-2-methyl-5-(pyridin-4-yl)thiophen-3-yl]acetate **(1f)** (75 mg, 0.17 mmol, 91%).

^{1}H NMR (400 MHz, $CDCl_3$) δ 1.02 (s, 3H), 1.04 (s, 3H), 1.20 (s, 9H), 1.22 (t, J = 7.1 Hz, 3H), 1.37-1.44 (m, 2H), 1.88-2.10 (m, 4H), 2.62 (s, 3H), 4.13-4.20 (m, 2H), 5.11 (s, 1H), 5.70-5.74 (m, 1H), 7.46 (dd, J = 1.6 Hz, J= 4.7 Hz, 2H), 8.50 (dd, J = 1.5 Hz, J= 4.7 Hz, 2H).

MS m/z ($[M+H]^+$) 442.

Step 7: preparation of intermediate 2-(*tert*-butoxy)-2-[4-(4,4-dimethylcyclohex-1-en-1-yl)-2-methyl-5-(pyridin-4-yl)thiophen-3-yl]acetic acid **(1g)**

Potassium hydroxide (9.5 mg, 0,17 mmol) was added to a solution of ethyl 2-(tert-butoxy)-2-[4-(4,4-dimethylcyclohex-1-en-1-yl)-2-methyl-5-(pyridin-4-yl)thiophen-3-yl]acetate **(1f)** (75 mg, 0.17 mmol) in a mixture of methanol (2.4 mL) and water (2.7 mL). The mixture was sonicated for 10 minutes and heated at 90°C for 7 hours. The mixture was concentrated to evaporate methanol *in vacuo*. The aqueous layer was acidified to pH 4-5 with a 1N hydrochloric acid aqueous solution and extracted three times with ethyl acetate. The organic layer was washed with brine, dried over sodium sulfate, filtered and concentrated *in vacuo*. The residue was purified by preparative TLC (dichloromethane/methanol 90/10) to give 2-(*tert*-butoxy)-2-[4-(4,4-dimethylcyclohex-1-en-1-yl)-2-methyl-5-(pyridin-4-yl)thiophen-3-yl]acetic acid **(1g)** (39 mg, 0.09 mmol, 55%).

^{1}H NMR (400 MHz, $CDCl_3$) δ 0.98 (s, 3H), 1.00 (s, 3H), 1.24 (s, 9H), 1.35-1.43 (m, 2H), 1.86-2.05 (m, 4H), 2.53 (s, 3H), 5.16-5.20 (bs, 1H), 5.64-5.73 (bs, 1H), 7.40-7.48 (bs, 2H), 8.50-8.58 (bs, 2H).

MS *m/z* ($[M-H]^-$) 412.

MUT-A: preparation of (2S)-2-(*tert*-butoxy)-2-[4-(4,4-dimethylcyclohex-1-en-1-yl)-2-methyl-5-(pyridin-4-yl)thiophen-3-yl]acetic acid

A sample of the compound 1g, 2-(*tert*-butoxy)-2-[4-(4,4-dimethylcyclohex-1-en-1-yl)-2-methyl-5-(pyridin-4-yl)thiophen-3-yl]acetic acid, has been loaded onto a Chiralcel OD column (20µm, 250 x 21.7mm) and eluted with a mixture of heptane/isopropanol/ acetic acid (95/5/0,1%) at ambient temperature and a flow rate of 21mL/min. Chiral purity has been assessed by chiral HPLC with a Chiralcel OD column (10µm, 250 x 4.6mm) eluted with a mixture of heptane/isopropanol/ acetic acid (95/5/0,1%) and a flow rate of 2mL/min. Fractions containing a single enantiomer with a retention time of 7.3 min (analytic HPLC) were combined and evaporated under reduced pressure to give the pure enantiomer, the (2S)-2-(*tert*-butoxy)-2-[4-(4,4-dimethylcyclohex-1-en-1-yl)-2-methyl-5-(pyridin-4-yl)thiophen-3-yl]acetic acid **(MUT-A)** with an enantiomeric excess of 98.04%.

^1H NMR (400 MHz, $CDCl_3$) δ 0.98 (s, 3H), 1.00 (s, 3H), 1.24 (s, 9H), 1.35-1.43 (m, 2H), 1.86-2.05 (m, 4H), 2.53 (s, 3H), 5.16-5.20 (bs, 1H), 5.64-5.73 (bs, 1H), 7.40-7.48 (bs, 2H), 8.50-8.58 (bs, 2H).

MS *m/z* ($[M-H]^-$) 412.

HTRF®-based IN-LEDGF interaction assay: IN-LEDGF HTRF® assay was performed in 384-well low volume black polystyrene plates (Corning) using IN-LEDGF assay buffer (25 mM Tris-HCl pH 7.4, 150 mM NaCl, 2 mM $MgCl_2$, 0.4 M KF, 0.1% Igepal CA-630, 0.1% bovine serum albumin, 1 mM DTT). 2 µL of 3-fold serial dilutions of inhibitory compound in 25% DMSO were preincubated for 30 min at room temperature with 8 µL of IN mixture (50 nM Flag-tagged IN, 17 nM XL_{665}-conjugated anti-Flag M2 monoclonal antibody). 10 µL of LEDGF mixture (60 nM His_6-tagged LEDGF/p75, 1.5 nM Terbium cryptate-labelled anti-His_6 monoclonal) were added and the plate was incubated for 2.5 h at room temperature before reading the time-resolved fluorescence in a PHERAstar Plus with HTRF module (excitation at 337 nm, dual emission at 620 nm and 667 nm). The HTRF ratio was converted to % inhibition and analyzed by fitting a sigmoidal dose-response equation with Hill slope to determine the IC_{50} of the compound.

HTRF®-based IN multimerization assay: IN-IN HTRF® assay was performed in 384-well low volume black polystyrene plates (Corning). 2 µL of 3-fold serial dilutions of inhibitory compound in 25% DMSO were preincubated for 30 min at room temperature with 4 µL of 125 nM Flag-IN dilution. 4 µL of 125 nM His_6-IN were added and the plate was incubated for 3 h at room temperature to allow IN subunit exchange and multimerisation. This step was performed in IN2 buffer (25 mM HEPES pH 7.4, 150 mM NaCl, 2 mM $MgCl_2$, 0.005% Tween-20, 0.1% bovine serum albumin, 1 mM DTT). 10 µL of revelation mixture (1.1 nM Europium cryptate-labelled monoclonal anti-Flag M2 antibody and 13 nM XL_{665}-labeled anti-His_6 monoclonal antibody in IN2 buffer supplemented with 0.8 M KF) were added and the plate was incubated for 2 h at room temperature before reading the time-resolved fluorescence in

a PHERAstar Plus with HTRF module (excitation at 337 nm, dual emission at 620 nm and 667 nm). The HTRF ratio was converted to % activation and analyzed by fitting a sigmoidal dose-response equation with Hill slope to determine the AC_{50} of the compound and the activation plateau.

Viral RNA isolation, Northern blot analyses, primer extension assays, RT activity assays: All these experiments were performed as described in detail in [22]. In all of them, MUT-A samples were run in parallel and collected simultaneously with BI-D-treated samples published previously [22], together with the same untreated control samples.

Cryo-electron microscopy of HIV particles: Cell culture supernatants containing HIV particles were fixed in 8% paraformaldehyde (EM grade) solubilized in a 4% cacodylate buffer. Virus particles were pelleted by ultracentrifugation in a SW32Ti rotor (Beckman) for 3 hours at 17 000 rpm together with 10 µL colloidal gold particles (Aurion, GaR 10) to better localize the pellet. Virus pellets were suspended in 50 µL Tris 50 mM pH 8 and 2.5 µL was applied to Quantifoil R2/2 holey carbon grids which were then plunge frozen into liquid ethane using a Vitrobot instrument (FEI). For data acquisition, grids were transferred to the cryo specimen holders on the cassette of the Titan Krios electron microscope equipped with a K2 camera (Gatan) and an energy filter (GIF). Images were acquired at 300 KV, using the EPU automatic data collection system (FEI) under low dose conditions at -6 µm defocus using the counting mode of the camera.

Determination of HIV-1 replication capacity: The replication capacity of wt and mutant viruses encoding amino acid substitutions in HIV integrase was evaluated in a short-term infectivity assay using TZM-bl reporter cell line. Briefly, 10^4 cells par well were added in duplicate into a 96-well culture plate in 50 µL of complete medium, Dulbecco's modified Eagle medium (DMEM) (Gibco) supplemented with 10% fetal bovine serum (Gibco), 1% penicillin-streptomycin (Gibco). Viral stocks for both wild-type and mutant viruses were normalized by p24, and serially diluted 5-fold from viral stock suspensions. 50 µL of virus

dilution was added to the cell culture. Virus and cells were co-cultured for 48 h, after which 100 μL of One-Glo® (Promega) reagent was added and luciferase activity measured in a luminometer as described in the manufacturer's instructions. The viral replication level was expressed as a percentage of relative light units (RLU) with reference to wild-type virus.

Supplementary Figure S1: 1H NMR spectrum of MUT-A

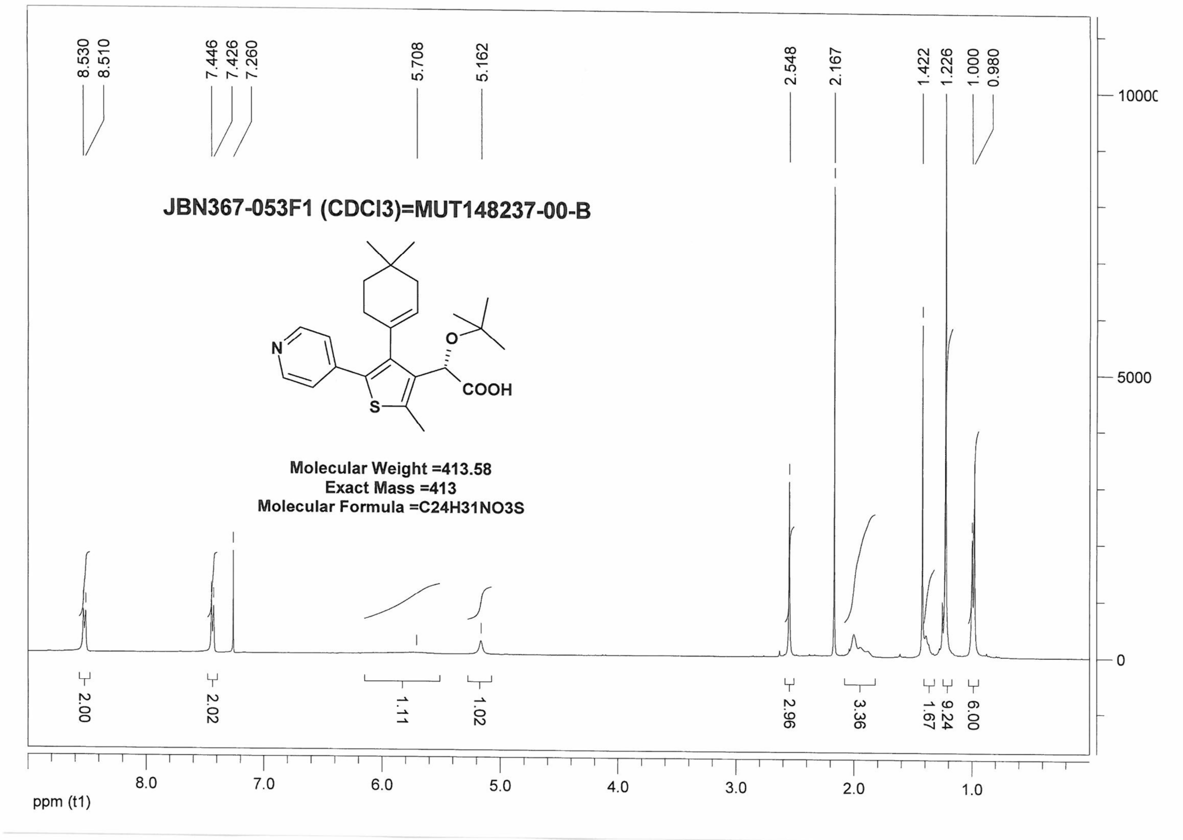

Supplementary Figure S2: Impact of MUT-A on HIV-1 replication and production. MUT-A-treated samples were run and collected in parallel with BI-D-treated viruses and untreated viruses used as controls that were previously published (van Bel et al, 2014). **A.** SupT1 cells were infected with HIV-1 LAI and cultured in the absence or presence of MUT-A (160 nM, 5x EC_{50}). **B.** Protocol of HIV-1 production upon transfection of 293T cells with HIV-1 LAI plasmid, cultured with or without MUT-A. **C.** Virus produced in the presence of absence of MUT-A was measured by CA-p24 ELISA after 48 h. Mock treated cells were transfected with control plasmid pBluescript-SK+. Average values with SD are shown (n=3). **D.** Protein content of HIV-1 produced in the presence or absence of MUT-A estimated by western blot using antibodies against Gag, Env, RT and IN viral proteins from HIV-1, and compared with virus produced in the presence of the protease inhibitor Saquinavir (SQV). **E.** The virus stocks produced in the presence or absence of MUT-A were used for infection of SupT1 T cells. No additional MUT-A was added during culturing. The CA-p24 level in the culture medium was monitored by ELISA.

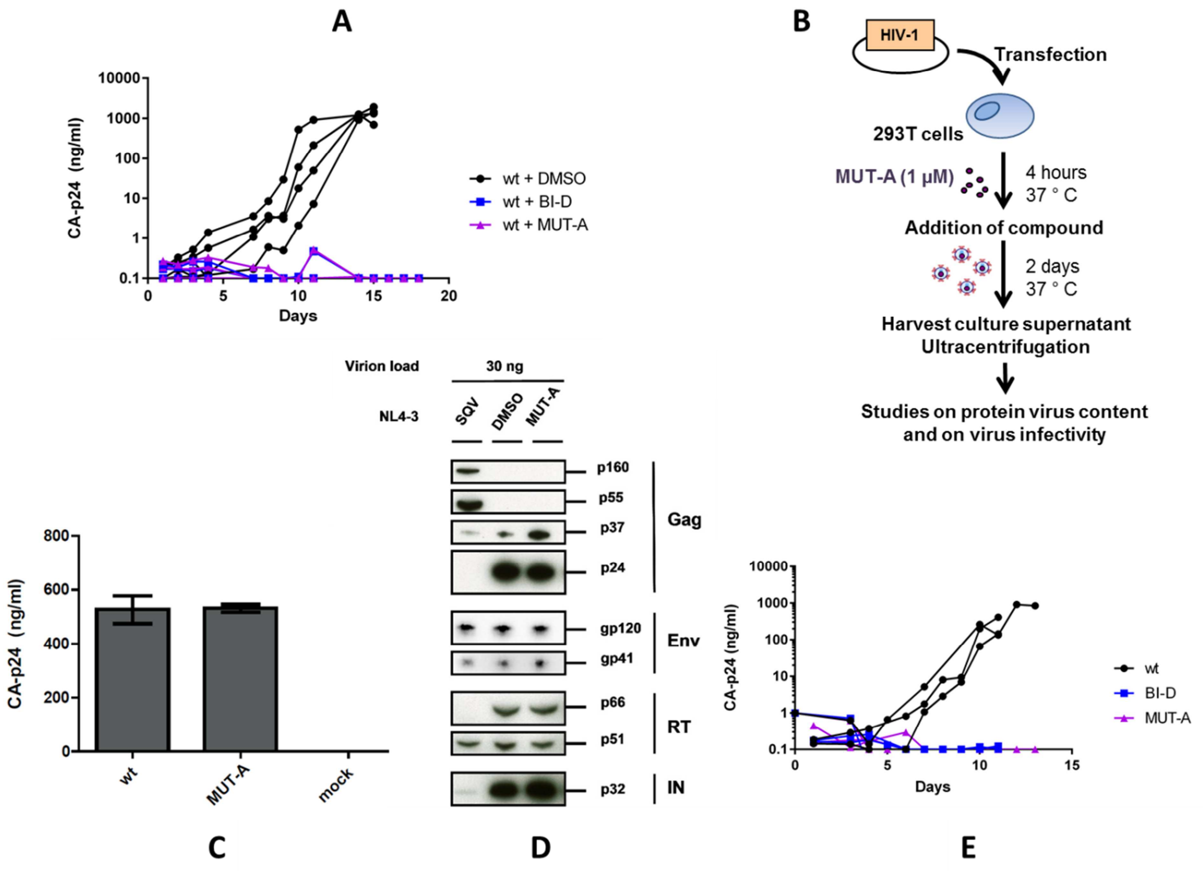

Supplementary Figure S3: Analysis of genomic RNA, Reverse transcriptase and tRNALys3 primer in MUT-A-treated HIV-1. A-D. HIV-1 RNA packaging and thermal stability of HIV-1 RNA dimers. MUT-A treated samples were run and collected in parallel with BI-D-treated and untreated samples used as controls that were previously published [22]; mock samples, RNA isolated from pBluescript-SK+ transfected cells. **A.** Denaturing northern blot to measure viral RNA packaging. **B.** Quantification of the 9-kb viral RNA from A. The average value (n=3) with SD is shown. **C.** Viral RNAs heated at increasing temperatures before analysis on a non-denaturing northern blot. The dimer and monomer positions are indicated. **D.** quantification to calculate the level of RNA dimerization at each temperature in C. **E. Activity of virus-extracted Reverse transcriptase.** Virus supernatant was incubated with an MS2 RNA template and dNTPs. The RT activity was determined by quantitation of the cDNA product by qPCR. Serial dilutions of AMV-RT were used for standard curve. Average values with SD are shown (n=2). Mock: supernatant of cells transfected with pBluescript-SK+. **F-J. tRNA^{lys3} occupancy of the HIV-1 RNA.** HIV-1 RNA-tRNA^{lys3} complexes were isolated from virions and analyzed by primer extension. Extension of tRNA^{lys3} primer yields a product of 257 nt. Extension of a heat-annealed CN1 primer results in a product of 151 nt. **F.** Both tRNA^{lys3} and CN1 primers were extended with exogenous HIV-1 RT (p66/51) and dNTPs. DNA products were run on a denaturing polyacrylamide gel. Lanes of one gel were merged. **G.** Schematic showing the primer extension assay. **H.** Quantification of the 257-nt band produced by tRNA^{lys3} primer. **I.** Quantification of the 151-nt band produced by the primer CN1. **J.** tRNA^{lys3} occupancy of the PBS was determined by calculating the tRNA to CN1 product ratio. The average value with SD is shown (n=4). Mock: supernatant of cells transfected with control plasmid pBluescript-SK+.

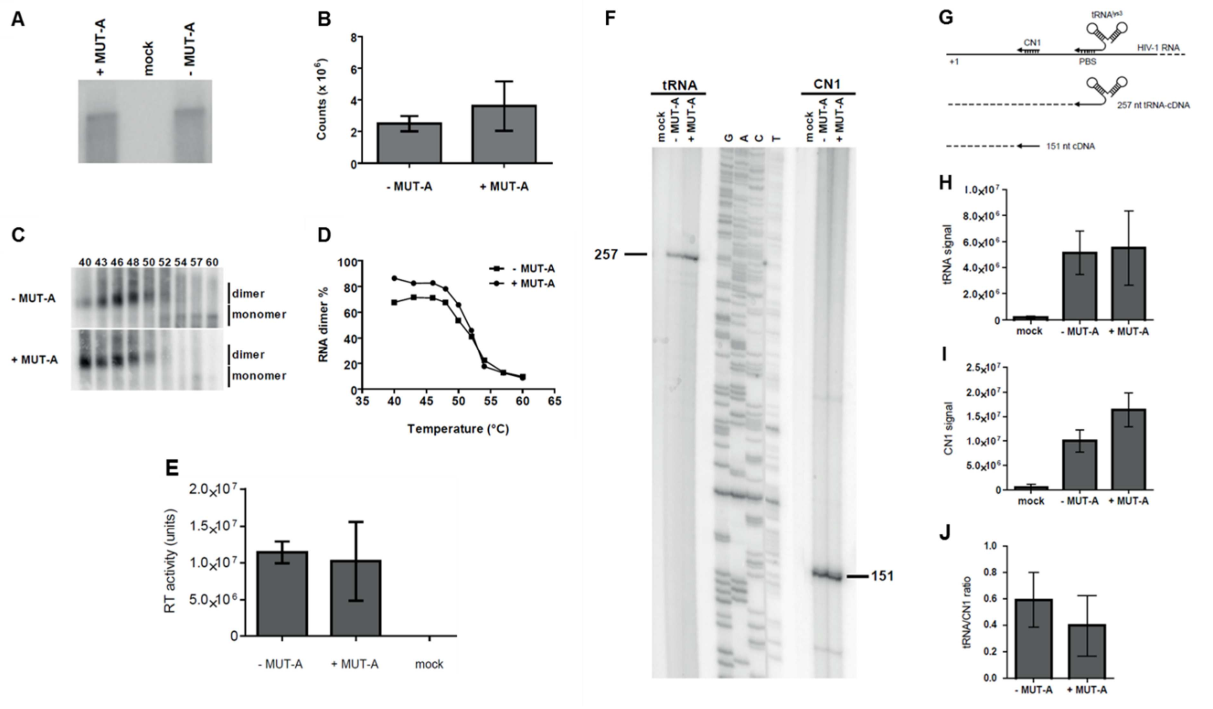
A
+ MUT-A
mock
- MUT-A
B
Counts (x 10^6)
- MUT-A
+ MUT-A
C
40 43 46 48 50 52 54 57 60
- MUT-A
+ MUT-A
dimer
monomer
D
RNA dimer %
- MUT-A
+ MUT-A
Temperature (°C)
E
RT activity (units)
- MUT-A
+ MUT-A
mock
F
tRNA
CN1
mock
- MUT-A
+ MUT-A
G
A
C
T
257
151
G
CN1
tRNAlys3
HIV-1 RNA
+1
PBS
257 nt tRNA-cDNA
151 nt cDNA
H
tRNA signal
I
CN1 signal
J
tRNA/CN1 ratio

Supplementary Figure S4: HIV-1 NL4-3 particles observed by cryo-EM. A. Viruses produced from 293T cells transfected with pNL4-3 in the presence of MUT-A. **B.** NL4-3 viruses produced in the absence of MUT-A. Red arrows indicate the formation of eccentric condensates, blue arrows indicate normal conical cores, and green arrows show non-conical cores. Scale bars are 100 nm.

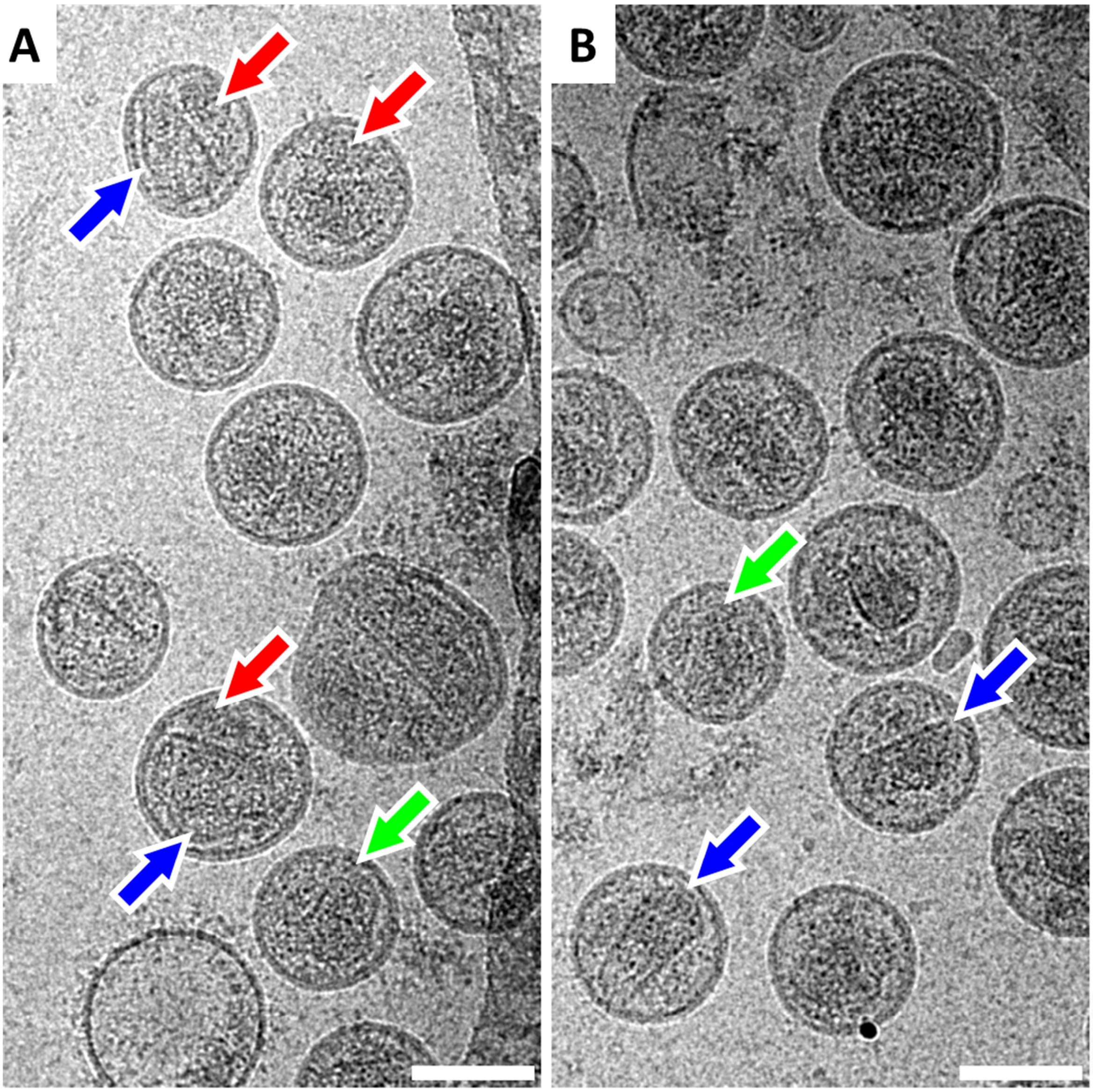

Supplementary Figure S5: Replicative capacity of HIV-1 NL4-3 viruses bearing resistance mutations to INLAIs or INSTIs. Results are expressed in percentage of replication capacity of wt NL4-3 (in yellow).

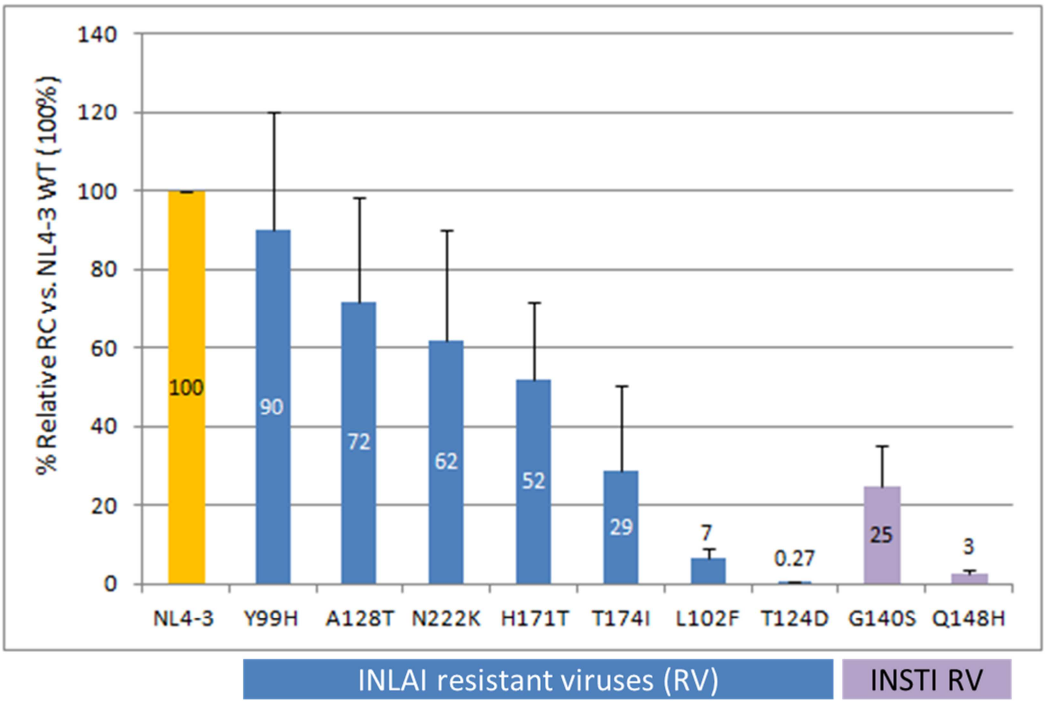

Supplementary Table S1: Immunoreactivity of HIV-1 NL4-3 produced in the presence of MUT-A, or Saquinavir, or after AT2 treatment, or in the presence of DMSO. Quantitative results of the antibody capture assay of native HIV-1 particles produced using the indicated antibodies. The capacity of the different antibodies to capture native HIV-1 particles was assessed by ELISA. HIV-1 particles retained by the antibodies were lysed and quantified by CA-p24 detection by ELISA.

		p24 concentration (ng/mL)															
		NL4-3 MUT-A				NL4-3 Saquinavir				NL4-3 AT-2				NL4-3 DMSO			
	Virus Dilution	Conc.1	Conc.2	Mean	SD	Conc.1	Conc.2	Mean	SD	Conc.1	Conc.2	Mean	SD	Conc.1	Conc.2	Mean	SD
B12	5 µg/mL	1.54	1.18	**1.36**	0.26	1.20	1.40	**1.30**	0.14	1.17	1.14	**1.16**	0.02	0.99	0.97	**0.98**	0.01
	1 µg/mL	0.82	0.71	**0.77**	0.08	1.00	0.77	**0.89**	0.16	0.88	0.92	**0.90**	0.03	0.57	0.50	**0.53**	0.04
	0.2 µg/mL	0.32	0.32	**0.32**	0.00	0.49	0.52	**0.51**	0.02	0.57	0.55	**0.56**	0.01	0.28	0.22	**0.25**	0.04
2G12	5 µg/mL	1.93	1.40	**1.67**	0.37	1.41	1.54	**1.47**	0.09	1.44	1.48	**1.46**	0.03	1.63	1.44	**1.53**	0.14
	1 µg/mL	1.15	0.94	**1.04**	0.15	0.99	1.06	**1.02**	0.04	1.50	1.45	**1.48**	0.04	0.72	0.99	**0.86**	0.19
	0.2 µg/mL	0.39	0.31	**0.35**	0.05	0.60	0.62	**0.61**	0.01	0.75	0.72	**0.73**	0.02	0.35	0.32	**0.34**	0.02
4D4	5 µg/mL	1.72	1.67	**1.69**	0.04	1.72	1.78	**1.75**	0.04	2.17	1.91	**2.04**	0.18	1.39	1.53	**1.46**	0.10
	1 µg/mL	1.34	0.96	**1.15**	0.27	1.30	1.33	**1.31**	0.02	1.76	1.53	**1.64**	0.16	1.13	1.10	**1.11**	0.02
	0.2 µg/mL	0.61	0.54	**0.58**	0.05	0.78	0.87	**0.83**	0.06	1.17	1.27	**1.22**	0.07	0.76	0.32	**0.54**	0.31
PGT121	5 µg/mL	1.53	1.28	**1.40**	0.18	0.61	0.71	**0.66**	0.07	0.38	0.53	**0.45**	0.11	1.44	0.68	**1.06**	0.53
	1 µg/mL	0.30	0.32	**0.31**	0.02	0.34	0.41	**0.37**	0.05	0.35	0.26	**0.30**	0.06	0.21	0.36	**0.29**	0.10
	0.2 µg/mL	0.17	0.15	**0.16**	0.01	0.27	0.34	**0.30**	0.05	0.40	0.40	**0.40**	0.00	0.20	0.16	**0.18**	0.03
IgG HIV-positive	1/100	0.81	0.83	**0.82**	0.01	0.88	0.73	**0.81**	0.11	1.29	1.39	**1.34**	0.07	0.83	1.01	**0.92**	0.13
	1/500	0.56	0.56	**0.56**	0.00	0.65	0.70	**0.67**	0.04	1.12	1.18	**1.15**	0.04	0.60	0.66	**0.63**	0.04
	1/2500	0.35	0.30	**0.32**	0.04	0.61	0.60	**0.60**	0.01	0.95	1.07	**1.01**	0.08	0.41	0.35	**0.38**	0.04
IgG HIV-negative	1/100	0.17	0.17	**0.17**	0.01	0.20	0.23	**0.22**	0.02	0.35	0.29	**0.32**	0.04	0.15	0.16	**0.16**	0.01
	1/500	0.12	0.14	**0.13**	0.01	0.17	0.26	**0.21**	0.06	0.30	0.32	**0.31**	0.01	0.10	0.13	**0.12**	0.02
	1/2500	0.16	0.14	**0.15**	0.01	0.27	0.26	**0.27**	0.01	0.33	0.34	**0.34**	0.00	0.11	0.12	**0.12**	0.01
Controls without IgG	Virus alone	0.14	0.16	**0.15**	0.01	0.14	0.16	**0.15**	0.01	0.36	0.35	**0.35**	0.00	0.12	0.14	**0.13**	0.01
	Buffer	0.11	0.06	**0.08**	0.04	0.11	0.06	**0.08**	0.04	0.07	0.06	**0.06**	0.01	0.05	0.06	**0.06**	0.00

Supplementary Figure S6: Results of virus immunocapture from Table S1 represented in bar graphs. The indicated antibodies were used at three different concentrations.

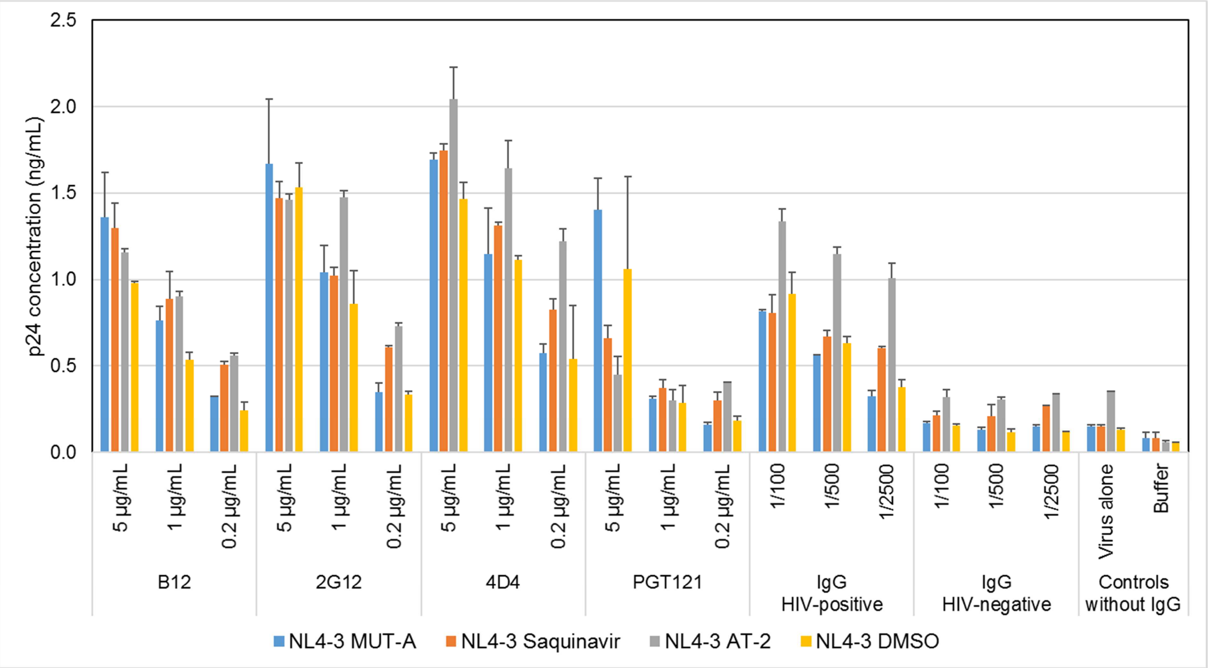

Supplement: Supplementary file 1 — Additional file 1. Supplementary methods. Chemical synthesis process of MUT-A,HTRF®-based IN-LEDGF interaction assay, HTRF®-based IN multimerization assay, Viral RNA isolation, Northern blot analyses, primer extension assays, RT activity assays, Cryo-electron microscopy of HIV particles, Determination of HIV-1 replication capacity. Figure S1: 1H NMR spectrum of MUT-A. Figure S2: Impact of MUT-A on HIV-1 replication and production. Figure S3: Analysis of genomic RNA, Reverse transcriptase and tRNALys3 primer in MUT-A-treated HIV-1. A-D. HIV-1 RNA packaging and thermal stability of HIV-1 RNA dimers. Figure S4: HIV-1 NL4-3 particles observed by cryo-EM. A. Figure S5: Replicative capacity of HIV-1 NL4-3 viruses bearing resistance mutations to INLAIs or INSTIs. Table S1: Immunoreactivity of HIV-1 NL4-3 produced in the presence of MUT-A, or Saquinavir, or after AT2 treatment, or in the presence of DMSO. Figure S6: Results of virus immunocapture from Table S1 represented in bar graphs. [file 12977_2017_373_MOESM1_ESM.pdf]
